# Lifespan Changes in Network Structure and Network Topology Dynamics During Rest and Auditory Oddball Performance

**DOI:** 10.3389/fnagi.2019.00138

**Published:** 2019-06-11

**Authors:** Viktor Müller, Viktor Jirsa, Dionysios Perdikis, Rita Sleimen-Malkoun, Timo von Oertzen, Ulman Lindenberger

**Affiliations:** ^1^Center for Lifespan Psychology, Max Planck Institute for Human Development, Berlin, Germany; ^2^Aix Marseille University, INSERM, INS, The Institut de Neurosciences des Systèmes, Marseille, France; ^3^Aix Marseille University, CNRS, ISM, The Institute of Movement Science, Marseille, France; ^4^Department of Psychology, Universität der Bundeswehr München, Neubiberg, Germany; ^5^Max Planck UCL Centre for Computational Psychiatry and Ageing Research, Berlin, Germany; ^6^Max Planck UCL Centre for Computational Psychiatry and Ageing Research, London, England

**Keywords:** lifespan changes, EEG, functional connectivity, directional coupling, network topology dynamics, graph-theoretical approach, resting state, auditory oddball performance

## Abstract

Behavioral and physiological evidence suggests that developmental changes lead to enhanced cortical differentiation and integration through maturation and learning, and that senescent changes during aging result in dedifferentiation and reduced cortical specialization of neural cell assemblies. We used electroencephalographic (EEG) recordings to evaluate network structure and network topology dynamics during rest with eyes closed and open, and during auditory oddball task across the lifespan. For this evaluation, we constructed a hyper-frequency network (HFN) based on within- and cross-frequency coupling (WFC and CFC, respectively) at 10 oscillation frequencies ranging between 2 and 20 Hz. We found that WFC increased monotonously across the lifespan, whereas CFC showed a U-shaped relationship. These changes in WFC and CFC strengths coevolve with changes in network structure and network topology dynamics, namely the magnitude of graph-theoretical topology measures increased linearly with age (except for characteristic path length, which is going shorter), while their standard deviation showed an inverse U-shaped relationship with a peak in young adults. Temporal as well as structural or nodal similarity of network topology (with some exceptions) seems to coincide with variability changes, i.e., stronger variability is related to higher similarity between consecutive time windows or nodes. Furthermore, network complexity measures showed different lifespan-related patterns, which depended on the balance of WFC and CFC strengths. Both variability and complexity of HFNs were strongly related to the perceptual speed scores. Finally, investigation of the modular organization of the networks revealed higher number of modules and stronger similarity of community structures across time in young adults as compared with children and older adults. We conclude that network variability and complexity measures reflect temporal and structural topology changes in the functional organization and reorganization of neuronal cell assemblies across the lifespan.

## Introduction

A growing body of evidence from electromagnetic and neuroimaging studies suggests that successful cognitive aging as well as maturation are determined by interactions both within and between largescale functional brain networks (Tsvetanov et al., 2016; Baum et al., 2017). Distinct cell assemblies communicate with each other to integrate single information flows into a common network (Müller et al., 2016). Much less, however, is known about lifespan changes in network architecture and network dynamics underlying complex interaction of spatially segregated cell assemblies and their integration into the brain system as a whole. One of the mechanisms underlying integration or communication between different cell assemblies might be the cross-frequency coupling (CFC) allowing accurate timing between different oscillatory rhythms (Jensen and Colgin, 2007; Jirsa and Müller, 2013). CFC can also promote selective and dynamic control of distributed functional cell assemblies (cf. Canolty and Knight, 2010; Canolty et al., 2010), and elevation of different dimensions of brain integration (Varela et al., 2001; Buzsáki and Draguhn, 2004). The so-called ‘communication through coherence' emphasizes synchronization within single bands or within-frequency coupling (WFC), although allows for inter-band modulation or CFC (Fries, 2015). Much less if everything is known about the complexity of such modulations or connections within and between brain networks and their changes across the lifespan. The present study aims at overcoming these limitations. For this purpose, we constructed hyper-frequency networks (HFNs) based on WFC and CFC, and analyzed variability, complexity, topology, and modular organization of the HFNs across the lifespan.

Recent literature suggests that brain (cortical) signal variability (i.e., transient temporal fluctuations in brain signal) could be used as a means to capture complex interactions between neuronal structures and cell assemblies, providing thus important information about network dynamics and brain states, as well as cognitive performance and mental activity (McIntosh et al., 2008, 2014; Deco et al., 2011; Garrett et al., 2011, 2015; Sleimen-Malkoun et al., 2015). It has been shown that older brains are less variable than younger brains (Garrett et al., 2011, 2015; Sleimen-Malkoun et al., 2015). More interestingly, brain signal variability seems to promote more accurate and less variable cognitive performance in development (McIntosh et al., 2008) and aging (Garrett et al., 2011). In this regard, it was also found that better-performing younger adults exhibit significantly greater brain variability and revealed vaster variability-based regional differentiation as compared with older, poorer performers (Garrett et al., 2011). Moreover, age-related differences in brain signal variability reflect aging-induced changes in dopaminergic neuromodulation (Garrett et al., 2015). Applying an entropy-based complexity measure (MSE, multi-scale entropy) to EEG and MEG signals, McIntosh et al. (2014) found that age-related changes in brain signal variability were timescale-dependent, with elderly's MSE curves showing higher entropy at fine scales and lower entropy at coarser scales. Using MSE and other variability or complexity measures to study fluctuations of cortical activity in young and older adults, Sleimen-Malkoun et al. (2015) found that in line with previous findings of McIntosh et al. (2014), the EEG signals displayed systematic age-related changes that were timescale-dependent and were more complex (variable) at shorter time scales, but less complex at longer scales in elderly as compared with younger adults.

In a number of studies, it has been demonstrated that methods and models derived from non-linear dynamics are suitable tools for describing brain variability or complexity dynamics in development and aging (Anokhin et al., 1996, 1999, 2000; Müller and Lindenberger, 2012). Specifically, it has been shown that non-linear dynamic complexity of EEG signals steadily increased with age during resting state (Anokhin et al., 1996; Müller and Lindenberger, 2012), accompanied by a steadily decrease in non-linear coupling (Müller and Lindenberger, 2012). During stimulus processing, a significant drop in complexity and a rise in non-linear coupling across the lifespan has been observed (Müller and Lindenberger, 2012). A negative correlation between EEG dynamic complexity and spectral coherence has also been found in the study of adolescence (Anokhin et al., 1999). Further, in a study by Anokhin et al. (2000), an overall increase in EEG dynamic complexity with brain maturation (between 7.5 and 16 years) both during resting state and performance of cognitive tasks was observed. Moreover, it has been shown that oscillatory brain activity and the corresponding phase synchronization dynamics are modulated during stimulus processing and task performance with a steadily increase of phase synchronization across the lifespan (Müller et al., 2009). Recently, Jirsa and Müller (2013) showed that CFC measures covering the interaction between different frequencies add another dimension to the understanding of complex neural dynamics of the frequency-specific neuronal networks. They also provided evidence that CFC, allowing accurate timing between different oscillatory rhythms, may be one of the mechanisms for a communication between different cell assemblies and integration or re-integration of different information flows. Furthermore, in our previous study, we suggested a new approach for construction of HFN based on WFC and CFC, and described changes in network topology dynamics (NTD) during resting state and auditory oddball performance (Müller et al., 2016). In contrast to previous research, this new approach allows to overcome at least two constrains: (i) HFN consider several frequencies integrated in a common network with all possible interactions between frequencies and electrodes, and (ii) NTD of HFN represents variability (and also similarity) not in single brain signals but examines structural and dynamic changes of brain networks (see also Müller and Lindenberger, 2014, for the use of the HFN approach in a hyper-brain study).

In the present work, we take a further step using the HFN approach to explore network dynamics by applying and introducing several indices of graph complexity providing further important information about the geometry or structure of complex networks beyond purely topological aspects. These graph complexity measures can be pooled into four different groups (Müller and Lindenberger, 2018): (i) spectral or energy measures: Graph Energy (*GE*) and Laplacian Energy (*LE*), (ii) product measures: efficiency complexity (*C_e_*) and graph index complexity (*Cr*), (iii) entropy measures: offdiagonal complexity (*OdC*) and partition entropy (*PE*), and (iv) dimensionality measures represented by correlation dimension of the network (*CDN*) and information dimension of the network (*IDN*). While most of these measures are well known in the literature (Gutman and Zhou, 2006; Claussen, 2007; Zhou et al., 2007; Kim and Wilhelm, 2008), *CDN* and *IDN* were here implemented based on correlation dimension algorithms for time series (Grassberger and Procaccia, 1983; Lutzenberger et al., 1992; Skinner et al., 1993) and its applications to complex networks (Daqing et al., 2011; Lacasa and Gómez-Gardeñes, 2013). These measures represent different aspects of network complexity (e.g., energy, entropy, dimensionality, etc.). We are confident that they would provide important information about the network complexity dynamics across the lifespan, which is a scarcely studied topic in the literature.

Here, we present EEG data obtained from 111 subjects across the lifespan. The conditions comprise rest with eyes closed (REC) and open (REO), and an auditory oddball task under an attended (AOT) and unattended (UOT) condition. Based on the above considerations, we predicted a more or less monotonous increase or inverted U-shaped lifespan-changes in variability and complexity of the networks, which will change their NTD patterns dependent on the measure used. We also expected more prominent changes in adults as compared to children, while we also presume developmental and aging-related differences. In addition, we expected significant associations between network topology and complexity measures and perceptual speed (PS) scores assessed in several tasks.

## Materials and Methods

The study design has been described previously (cf. Müller et al., 2009). Here, we investigated the same group of participants. We also used (but in different context) the data showing their performance on perceptual speed tasks. However, EEG analyses were carried out on different segments or segment lengths, and by using distinct algorithms based on synchronization across time within and between different frequencies.

### Participants

All participants were volunteers, right-handed, had no reported history of head or neurological disorders, and none were on medication (cf. Müller et al., 2009). The effective sample consisted of 24 younger children (YC, mean age = 9.9, *SD* = 0.6, age range = 9.0–10.8 years, 13 females), 28 older children (OC, mean age = 12.0, *SD* = 0.6, age range = 11.0–12.8 years, 14 females), 31 younger adults (YA, mean age = 22.7, *SD* = 1.6, age range = 18.8–25.1 years, 14 females), and 28 older adults (OA, mean age = 67.8, *SD* = 3.0, age range = 63.9–74.5 years, 14 females). Participants of all ages including children were able to sustain their attention for the entire duration of the experiment. The study has been approved by the ethics committee of Saarland University and has therefore been performed in accordance with the ethical standards laid down in the 1964 Declaration of Helsinki. All subjects gave their written informed consent prior to their inclusion in the study.

### Psychological Assessment

Psychological assessment was carried out on a different day preceding the EEG session. For psychological assessment, the cognitive battery of the Berlin Aging Study (BASE; Baltes and Mayer, 1999) was used. Three tests from this battery—Digit Symbol Substitution (DSS), Digit Letter Substitution (DLS), and Identical Pictures (IP)—are marker tests of perceptual speed (PS) and were selected for correlational analysis of relations with electrophysiological data. The materials and procedural details of the cognitive battery have been described elsewhere (Lindenberger et al., 1993; Müller and Lindenberger, 2012).

Briefly, the Wechsler (1955) version of the DSS test was used. We presented the participants with a coding key pairing 9 numbers (1 through 9) with 9 symbols. Printed under the coding key were rows of randomly ordered numbers with empty boxes below. Participants had to write as many symbols as possible into the empty boxes based on the digit–symbol associations specified in the coding key within 90 s. The number of correctly completed items represented the outcome measure.

The DLS test closely resembles the DSS except that subjects had to name letters instead of writing symbols. The test consisted of a total of 21 sheets. Each sheet contained six digits with a question mark underneath. Moving from left to right, subjects had to name the letters that corresponded to the digits. Testing lasted for 3 min, with scores being taken after each minute. The score used here is based on the total number of correct responses after 3 min.

In the IP test, a total of 32 items was presented. For each item, a target figure was presented in the upper half of the screen, and five response alternatives were presented in the lower half. Participants had to touch the correct (identical) figure in the lower half as fast as possible. Before the test phase, instructions and three practice items were given. Testing was terminated automatically after 80 s. The score refers to the number of correct responses (cf. Müller and Lindenberger, 2012).

### Procedure

The EEG measurement began with a 3 min relaxation phase (1.5 min with eyes closed and 1.5 min with eyes open). During the recording, the subjects sat in a chair in a relaxed position in an electrically shielded room. The rest phases were followed by the auditory oddball task. During the oddball task, which was carried out with eyes closed, the participants heard two different types of tone pips: a 1,000 Hz tone played frequently to form the standard stimulus and an 800 Hz tone played only occasionally to form the deviant stimulus. The standard and deviant stimuli were presented binaurally (with a probability of 0.8 and 0.2 for the standard and deviant stimuli, respectively) through headphones (Sony DJ MDR-V300) at 70 dB SPL for a duration of 70 ms (including a 10-ms rise and fall period). The stimuli were generated using the Audacity 1.2.4 software. The inter-stimulus interval (ITI) was uniformly chosen at random between 1,200 and 1,500 ms. Two different experimental conditions were used: passive listening (unattended) and active counting (attended). For the first condition, the subjects were simply asked to listen to the tone pips without any response, whereas, for the second condition, the subjects were asked to listen to the stimuli and count the number of deviant tones. Each experimental condition contained 152 standard tones and 38 deviant tones presented in a pseudo-random order fixed for all participants. The conditions were always presented in the same order, with the passive listening condition followed by the active counting condition in order to facilitate the interpretation of between-person differences (see Müller et al., 2009 for details).

### EEG Recordings and Preprocessing

The electroencephalogram (EEG) was recorded from 58 Ag/AgCl electrodes using an elastic cap (Electrocap International) with a sampling rate of 500 Hz in a frequency band ranging between 0.5 and 100 Hz. The left mastoid was used as a reference and the right mastoid was recorded as an active channel. The data were also re-referenced off-line to an average of the left and right mastoids for further analysis. The electrodes were placed according to the international 10–10 system. The vertical and horizontal electrooculograms (EOG) were recorded for control of eye blinks and eye movements. Signals were digitally filtered off-line (Butterworth zero phase filters 1–100 Hz, slope 12 dB/octave; notch filter 50 Hz). Eye movement correction was accomplished by independent component analysis (Vigário, 1997) using BrainVision Analyzer (Brain Products, Gilching, Germany). Thereafter, artifacts from head and body movements were rejected by visual inspection. Finally, data were downsampled to a sampling rate of 250 Hz, segmented in artifact free 10 s segments (i.e., comprising *N_t_* = 2,500 data points each), and normalized within segments before further analysis.

### Within- and Cross-Frequency Coupling

To investigate phase coupling in a directed and frequency-resolved manner (cf. Müller et al., 2016), we used an *Integrative Coupling Index* (*ICI*) that was calculated as described elsewhere (Müller and Lindenberger, 2011; Müller et al., 2016). For this calculation, we first applied an analytic or complex-valued Morlet wavelet transform computing the instantaneous phase in the frequency range from 0 to 20 Hz. The complex mother Morlet wavelet, also called Gabor wavelet, has a Gaussian shape around its central frequency *f*:

(1) $$\begin{array}{l} w\left(t,f\right)={\left(\sigma^{2}\pi \right)}^{-1/4}e^{\left(\left(-t^{2}/2\sigma^{2}\right)+3/2\pi jft\right)},j=\sqrt{-1} \end{array}$$

in which σ is the standard deviation of the Gaussian envelope of the mother wavelet. The wavelet coefficients were calculated with a time step of 5 leading to a time resolution of 20 ms and frequency resolution of 0.125 Hz. In order to identify the phase relations within and between any two channels or frequencies, the instantaneous phase difference was computed from the wavelet coefficients for all possible electrode and frequency pairs. On the basis of instantaneous phases for two signals (*X* and *Y*) given as Φ_*X*_(*f_m_,t*) = *arg*[ϕ_*X*_(*f_m_,t*)] and Φ_*Y*_(*f_n_,t*) = *arg*[ϕ_*Y*_(*f_n_,t*)], respectively, with *arg* denoting an argument of the complex number, and ϕ_*X*_ and ϕ_*Y*_ being complex numbers, the *n:m* phase synchronization between two oscillations at the frequencies *f_m_* and *f_n_* was determined. The generalized phase difference (ΔΦ) according to equality *n*·*f_m_* = *m*·*f_n_* was calculated by:

(2) $$\begin{array}{l} \Delta \Phi \left(f_{m},f_{n},t\right)=n\cdot \Phi \left(f_{m},t\right)-m\cdot \Phi \left(f_{n},t\right),\text{mod}2\pi \end{array}$$

In the case of WFC with *f_m_* = *f_n_*, the phase difference ΔΦ is calculated in the same way by setting *m* = *n* = 1. Phase differences and corresponding phase synchronization measures were determined for 10 different frequencies of interest (FOI): 2, 4, 6, 8, 10, 12, 14, 16, 18, and 20 Hz, resulting in different frequency relations, such as 1:2, 1:3, 1:4, 2:3, 3:4 etc.

Thereafter, *ICI* was determined between all possible electrode and frequency pairs. *ICI* ranges between 0 and 1 and is an asymmetric coupling measure (i.e., *ICI*_XY_ ≠ *ICI*_YX_), indicating the relative extent of the positive shift in phase difference between two signals. To investigate the dynamic changes in phase synchronization and network topology (see below), we calculated phase coupling using moving time window of 2,000 ms width and 100 ms time delay. Overall, within a segment of 10-s duration, coupling measures across 81 time widows were collected by this shifting procedure.

### Network Construction

*ICI* values were used to construct a connectivity matrix or a graph representing the network properties, where each node is defined as a combination of electrode location and oscillation frequency. This means that the same electrode site at the 10 FOI represents 10 different nodes that communicate with other nodes at the same or different frequencies. There were 580 nodes altogether (58 electrodes × 10 frequency bins = 580) in the common HFN (cf. Müller et al., 2016). As *ICI* is a directed measure, the constructed networks were directed weighted graphs.

#### Threshold Determination

In general, the choice of a threshold plays an important and non-trivial role in network construction, but is necessarily always arbitrary. At least two issues appear important for us in this study: (i) the connectivity measures should not occur by chance, and (ii) the networks changing in time should have the same threshold, which correspond to a high sparsity level. For determining the network properties across the different time windows, we set the connectivity threshold to 0.26, which was always higher than the significance level determined by the surrogate data procedure (see below). At this threshold, the cost level of the networks (ratio of the number of actual connections divided by the maximum possible number of connections in the network) was approximately 20%, corresponding to high sparsity of the resulting networks and allowing more accurate examination of the network topology across conditions and lifespan samples. Being aware that an absolute threshold could cause slightly different cost levels, we calculated the costs in order to use them as covariates.

#### Surrogate Data Procedure

Surrogate data were created in two ways: (1) by random permutations of the original time series, and (2) by phase permutation of the time series. The latter surrogate data procedure involved: (a) computing the amplitude and phase spectrum of a real signal using a Fourier transformation; (b) phase shuffling, whereby the phase values of the original spectrum are used in random order and the sorted values of the surrogate sequence are replaced by the corresponding sorted values of the reference sequence; and (c) inverse Fourier transformation back to the time domain. In this way, the real and the surrogate data retain the same power spectrum but a different time course. Thereafter, we applied a bootstrapping procedure with 1,000 resamples of the coupling measures resulting from the surrogate data sets and determined the significance level (*p* < 0.001) as the bootstrapping mean plus the confidence interval. The chosen threshold of 0.26 was always higher than the determined significance level in both surrogate data procedures and corresponded to a relatively high sparsity level, i.e., it matched both of our criteria (see above).

### Network Topology Metrics and Dynamics

Since for our further analyses and calculation of spatiotemporal NTD metrics we were interested in nodal network characteristics, we first determined all the GTA measures described below for each node and time window separately. Thereafter, the nodal GTA measures were averaged across time windows or nodes with regard to temporal or structural dynamics (see details below), whereby the set size was equal to 81 or 580, respectively.

#### Strengths

As *ICI* is a directed weighted measure, we obtained the nodes' in- and out-strengths, whereby the in-strength is defined as the sum of weights of all incoming connections (*w_ji_*), $S_{in}=\underset{j\in N}{\sum }w_{ji}$, and the out-strength is the sum of weights of all outgoing connections (*w_ij_*), $S_{out}=\underset{j\in N}{\sum }w_{ij}$. Note that strengths were first determined for each node separately and then averaged across time or nodes (see below). The overall strength (*S*) was calculated as a sum of in- and out-strengths: *S* = *S_in_* + *S_out_*. The overall strengths were calculated for WFC and CFC separately, to investigate their influence on network complexity and network dynamics.

#### Clustering Coefficient and Characteristic Path Length

For an individual node, the clustering coefficient (*CC*) is defined as the proportion of the number of existing neighbor–neighbor connections to the total number of possible connections within its neighborhood. In the case of a weighted directed graph, the mean *CC* is calculated as (Fagiolo, 2007):

(3) $$\begin{array}{l} CC=\frac{1}{m}\underset{i\in N}{\sum }CC_{i}^{wd}\\ \;=\frac{1}{m}\underset{i\in N}{\sum }\frac{t_{i}^{wd}}{\left(k_{i}^{out}+k_{i}^{in}\right)\left(k_{i}^{out}+k_{i}^{in}-1\right)-2\sum_{j\in N}a_{ij}a_{ji}} \end{array}$$

with $CC_{i}^{wd}$ being the nodal *CC* and $t_{i}^{wd}=\;\frac{1}{2}\underset{j,h\in N}{\sum }{\left[\left({w_{ij}}^{1/3}{w_{ih}}^{1/3}{w_{jh}}^{1/3}\right)+\left({w_{ji}}^{1/3}{w_{hi}}^{1/3}{w_{hj}}^{1/3}\right)\right]}^{3}$ being the number of weighted directed triangles around a node *i*; $k_{i}^{in}$ and $k_{i}^{out}$ are in- and out-degrees of the node *i*, *a_ji_*, and *a_ij_* are directed links of the adjacency matrix, *N* denotes the network size or the number of nodes, and *m* is the set size used for averaging. The *CC* measures the cliquishness of a typical neighborhood and is thus a measure of network segregation.

Another important measure is the characteristic path length (*CPL*). As our networks are directed weighted graphs, the weight and direction of the links must be considered. The input matrix is then a mapping from weight to length (i.e., a weight inversion), and the distance $d_{ij}^{wd}$ is the minimal weighted directed distance between the nodes *i* and *j*. *CPL* was determined as (Watts and Strogatz, 1998):

(4) $$\begin{array}{l} CPL=\frac{1}{m}\underset{i\in N}{\sum }L_{i}^{wd}=\frac{1}{m}\underset{i\in N}{\sum }\frac{\sum_{j\in N,j\neq i}d_{ij}^{wd}}{n-1} \end{array}$$

where $L_{i}^{wd}$ denotes the nodal or average shortest path length from node *i* to all other nodes in the network, *n* is the number of nodes, and *m* is the set size used for averaging.

#### Local and Global Efficiency

Local efficiency *(E_local_)* is similar to the *CC* and is calculated as the harmonic mean of neighbor-neighbor distances (Latora and Marchiori, 2001):

(5) $$\begin{array}{l} E_{local}=\frac{1}{m}\underset{i\in N}{\sum }E_{local\left(i\right)}^{wd}\\ \;=\frac{1}{m}\underset{i\in N}{\sum }\frac{t_{e}^{wd}}{\left(k_{i}^{out}+k_{i}^{in}\right)\left(k_{i}^{out}+k_{i}^{in}-1\right)-2\sum_{j\in N}a_{ij}a_{ji}} \end{array}$$

with $t_{e}^{wd}=\frac{1}{2}\sum_{j,h\in N,j\neq i}\left({w^{1/3}}_{ij}+{w^{1/3}}_{ji}\right)\left({w^{1/3}}_{ih}+{w^{1/3}}_{hi}\right)$ $\left({\left({\left[d_{jh}^{wd}\left(N_{i}\right)\right]}^{-1}\right)}^{1/3}+{\left({\left[d_{hj}^{wd}\left(N_{i}\right)\right]}^{-1}\right)}^{1/3}\right)$, where *N_i_* denotes the subgraph comprising all nodes that are immediate neighbors of the node *i*, $k_{i}^{in}$, and $k_{i}^{out}$ are in- and out-degrees of the node *i*, *a*_*ij*_ and *a*_*ji*_ are directed links of the adjacency matrix, *n* is the number of nodes, and *m* is the set size used for averaging. Thus, *E_local_* of node *i* is defined with respect to the subgraph comprising all of *i*'s neighbors, after removal of node i and its incident edges (Latora and Marchiori, 2001). Like *CC*, *E_local_* is a measure of the segregation of a network, indicating efficiency of information transfer in the immediate neighborhood of each node and showing how fault-tolerant the system is.

Global efficiency *(E_global_)* is normally defined as the average inverse shortest path length, and is calculated by (Latora and Marchiori, 2001):

(6) $$\begin{array}{l} E_{global}=\frac{1}{m}\underset{i\in N}{\sum }E_{global\left(i\right)}^{wd}=\frac{1}{m}\underset{i\in N}{\sum }\frac{{\sum_{j\in N,j\neq i}\left(d_{ij}^{wd}\right)}^{-1}}{n-1} \end{array}$$

where $E_{global\left(i\right)}^{wd}$ is the nodal efficiency, which is defined as the normalized sum of the reciprocal of the shortest path lengths or distances $d_{ij}^{wd}$ from a given node (*i*) to all other nodes in the network, *n* is the number of nodes, and *m* is the set size used for averaging. Like *CPL*, *E_global_* is a measure of the integration of a network, but whereas *CPL* is primarily influenced by long paths, *E_global_* is primarily influenced by short ones.

#### Network Topology Dynamics

**Figure 1:**
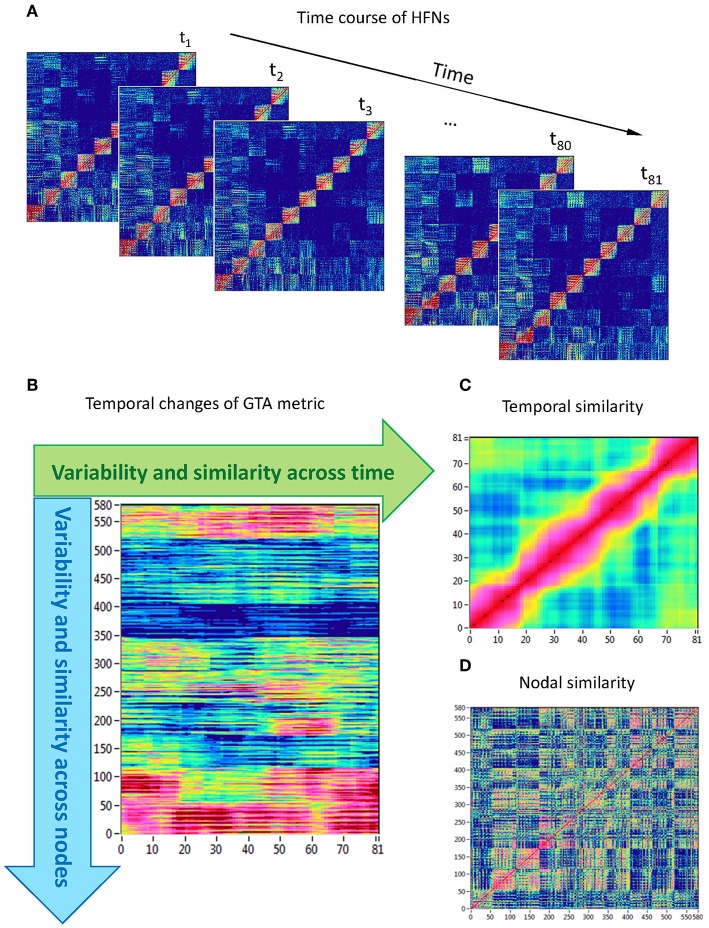
Determination of HFN dynamics with temporal and nodal variability and similarity. **(A)** Time course of HFNs calculated in the 10-s segment using a moving time window of 2,000 ms and time delay of 100 ms (81 time windows in total). **(B)** After the calculation of different GTA metrics for HFN in each time window, a *time windows* × *nodes* matrix (81 × 580) was constructed. In this matrix, mean (*M*), and standard deviation (*SD*) were determined both across time and across nodes. In addition, temporal and nodal similarity were determined also both across time and across nodes. **(C)** Temporal similarity matrix was built by calculation of Pearson's product correlation among the consecutive vertical lines in previous 81 × 580 matrix (each line represents 580 nodes of a GTA metric in corresponding time window). In the temporal similarity matrix, average strength was determined as a global temporal similarity index. **(D)** Nodal similarity matrix was built by calculation of Pearson's product correlation among the consecutive horizontal lines in previous 81 × 580 matrix (each line represents time course of a single node of a GTA metric). In the nodal similarity matrix, average strength was determined as a global nodal similarity index. HFN, hyper-frequency network; GTA, graph-theoretical approach. [Modified from Müller et al. (2016)].

Network topology given by the GTA measures specified above changes across time. To capture the spatiotemporal NTD, we calculated for each time window and each HFN node the six GTA metrics specified above (Figure 1A), then build for each GTA metric a ‘nodes × time windows' matrix (580 × 81, Figure 1B), and calculated the means (*M*) and standard deviations (*SD*) both across time windows (*tSD*) and across nodes (*nSD*). For statistical evaluation and in order to achieve a global measure for dynamic and structural variability, means and SDs were further averaged across nodes and time points, respectively. Thereafter, we determined *temporal network similarity*, i.e., correlation among time windows resulting in an 81 × 81 matrix (Figure 1C), and spatial or *nodal network similarity*, i.e., correlation among nodes resulting in a 580 × 580 matrix (Figure 1D). Both similarity measures were determined by Pearson's product correlation. Resulting correlation matrices were used for determination of overall temporal and nodal network similarity. For these purposes, we calculated average strength in resulting correlation matrices. Since nodal network similarity contained positive as well as negative values, we calculated two means or average strengths for positive and negative correlation values, respectively.

### Network Complexity Measures

We used different graph complexity measures to investigate network complexity dynamics. First, we determined *Graph Energy* (*GE*) and *Laplacian Energy* (*LE*) of a network. The *GE* is defined as (Gutman and Zhou, 2006):

(7) $$\begin{array}{l} GE=\overset{n}{\underset{i=1}{\sum }}|\lambda_{i}| \end{array}$$

where λ_*i*_ = λ_1_, λ_2_, …, λ_*n*_ are the eigenvalues of the weighted directed adjacency matrix. *GE* was normalized by dividing by 2*n*.

The *LE* was determined by the formula (Gutman and Zhou, 2006):

(8) $$\begin{array}{l} LE=\overset{n}{\underset{i=1}{\sum }}|\mu_{i}-\frac{2m}{n}| \end{array}$$

where μ_*i*_ = μ_1_, μ_2_, …, μ_*n*_ are eigenvalues of the Laplacian matrix, and *n* and *m* are the numbers of vertices and edges, respectively. *LE* was then normalized by dividing by the maximal number of edges: *n*(*n* – 1).

Next, we determined *graph index complexity* *C_r_* based on the largest eigenvalue of a graph called index *r*, which fulfills the inequality 2cos(π / (*n* + 1)) ≤ *r* ≤ *n* – 1. *C_r_* complexity is defined then as (Kim and Wilhelm, 2008):

(9) $$\begin{array}{l} C_{r}=4c_{r}\left(1-c_{r}\right)\text{ with }c_{r}=\frac{r-2\cos\frac{\pi }{n+1}}{n-1-2\cos\frac{\pi }{n+1}} \end{array}$$

where 0 ≤ *C_r_* ≤ 1.

*Efficiency complexity* *C_e_* was determined as (Kim and Wilhelm, 2008):

(10) $$\begin{array}{l} C_{e}=4\left(\frac{E-E_{path}}{1-E_{path}}\right)\left(1-\frac{E-E_{path}}{1-E_{path}}\right) \end{array}$$

where *E* is the global efficiency of a graph and *E_part_* is efficiency of the least efficient graph, which is determined by:

(11) $$\begin{array}{l} E_{path}=\frac{2}{n\left(n-1\right)}\overset{n-1}{\underset{i=1}{\sum }}\left(\frac{n-i}{i}\right) \end{array}$$

*Off-diagonal complexity* (*OdC*) was calculated by the formula (Claussen, 2007):

(12) $$\begin{array}{l} OdC=-\overset{M-1}{\underset{i=1}{\sum }}\frac{L_{i}}{m}\text{log}\left(\frac{L_{i}}{m}\right) \end{array}$$

where *L_i_* is the sum of entries in the *i^th^* diagonal. We adapted this measure for directed weighted graph by calculating upper and lower weighted diagonal entries, and normalized it through dividing by ln(n-1).

*Partition entropy* (*PE*) is the entropy of the distribution of community sizes when the nodes are separated into communities (Onnela et al., 2012):

(13) $$\begin{array}{l} PE=-\overset{N}{\underset{i=1}{\sum }}\frac{C_{i}}{n}\log\left(\frac{C_{i}}{n}\right) \end{array}$$

where *C_i_* is a size or the number of nodes in the community *i* and n is the number of nodes in the network. *C_i_* was determined using the modularity optimization method as described below.

Further, we estimated complexity of the network with regard to its dimensionality. We used a correlation dimension algorithm similar to the one introduced by Grassberger and Procaccia (1983) for time delay embedding (cf., Lacasa and Gómez-Gardeñes, 2013). As a first step, we calculated weighted distances of the network. Using the distance matrix, we calculated the correlation integral or the correlation sum function by comparing the distances with some scalar r:

(14) $$\begin{array}{l} C_{m}\left(r\right)=\frac{2}{m\left(m-1\right)}\overset{m}{\underset{i,j=1}{\sum }}\Theta \left(r-d_{ij}\right) \end{array}$$

where Θ is Heaviside step function, *m* is embedding dimension, and *d_ij_* is the distance between nodes *i* and *j*; scalar or preselected distance *r* varies with a logarithmic step (100 steps were used for calculation here) in the range between the smallest and the largest distance in the given network. The embedding dimension *m* was equal to the number of nodes in the network. The *correlation dimension of the network* (*CDN*) was determined as a slope in double logarithmic coordinates when plotting *C_m_*(*r*) as function of *r*:

(15) $$\begin{array}{l} CDN=\underset{r\to 0}{\text{lim}}\frac{\text{lg}\left(C_{m}\left(r\right)\right)}{\text{lg}\left(r\right)} \end{array}$$

For determination of the slope or *CDN*, the region between the 10th and 30th *r*-value was used, where the dependence curve (*C_m_*(*r*) vs. *r*) showed clear linear trend.

Using similar partition procedure, the *information dimension of the network* (*IDN*) can be determined by:

(16) $$\begin{array}{l} IDN=-\overset{M\left(r\right)}{\underset{i=1}{\sum }}\frac{C_{i}}{N}\text{log}\left(\frac{C_{i}}{N}\right) \end{array}$$

where *C_i_* is the number of nodes in the partition *i, M(r)* is the number of the partitions with a partition range *r*, and *N* indicates the overall number of nodes used in the calculation with *N* = *m*(*n-1*). As previous, the embedding dimension *m* was equal to the number of nodes in the network. There were 100 partitions (i.e., *M(r)* = 100) in the range between the smallest and the largest distance in the given network.

**Figure 2:**
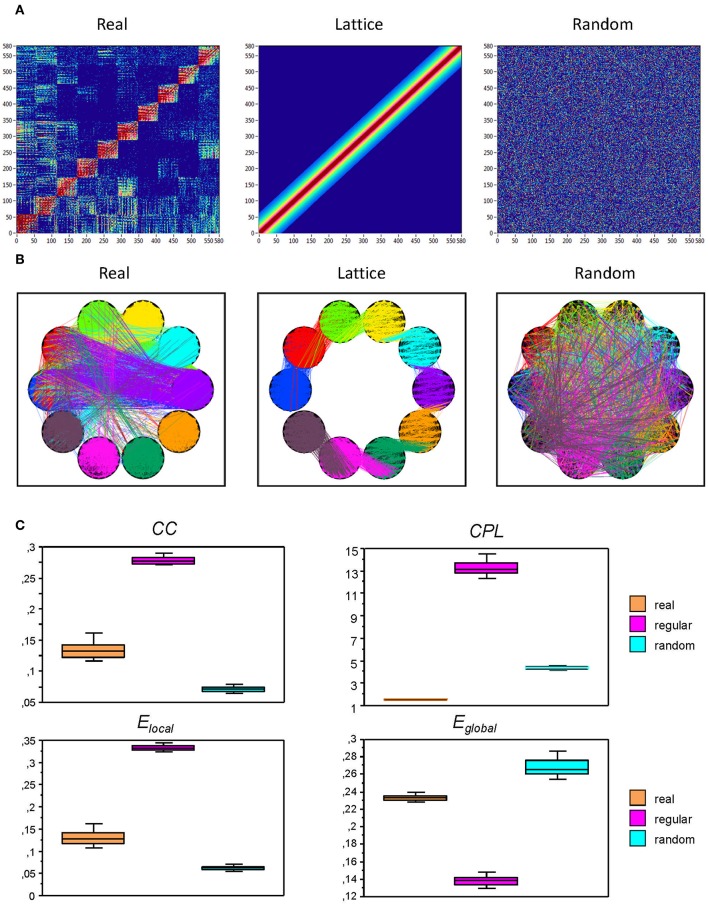
Representation of real, regular (lattice), and random networks. **(A)** In the real network (left), the nodes are organized by electrode location and oscillation frequency. The lattice network (middle) was configured by the randomization of the edges in the real network and consecutive redistribution in such a way that the strongest edges lay close to the main diagonal. The random network (right) was configured by the randomization of the edges only. The lattice and random networks were reconstructed in such a way that they have the same number of nodes and edges as the initial real network, but are characterized by ring (lattice) or random network topology. **(B)** The same networks as in **(A)**, represented in the form of brain maps including WFC (within-frequency coupling) and CFC (cross-frequency coupling). **(C)** Box plots of the GTA metric for the real and control (regular and random) networks. Box plots of clustering coefficient (*CC*), characteristic path length (*CPL*), local efficiency (*E_local_*), and global efficiency (*E_global_*) are presented. For this presentation, data were determined for attended oddball task (AOT) condition and averaged across all subjects.

To investigate the network topology and network complexity of the real networks, we constructed regular (lattice) and random networks that have the same number of nodes and edges as our real networks (see Figure 2). For these purposes, we first randomized the edges in the real network to achieve a random network; lattice network was configured like random network, but in addition, edges were redistributed after an initial random permutation such that they lay close to the main diagonal with increasing order of their weights. Lattice network reconstructed in such a way has the same number of nodes and edges as the initial real network but is characterized by ring or lattice topology incorporating nearest-neighbor connectivity (Sporns et al., 2007). Control networks (i.e., regular and random networks) were constructed for all subjects for AOT condition only.

### Modular Organization of the Networks and Its Dynamic Changes

To further investigate the topological properties of the HFNs, community structures and modularity index (*Q*) were determined. For this calculation, the modularity optimization method for directed graphs that is implemented in the Brain Connectivity Toolbox (Rubinov and Sporns, 2010) was used. The optimal community structure is a subdivision of the network into non-overlapping groups of nodes or communities in a way that maximizes the number of within-module edges and minimizes the number of between-module edges. The modularity (*Q*) is a statistic that quantifies the degree to which the network may be subdivided into such clearly delineated groups or modules. For directed networks, this is given by the formula (Leicht and Newman, 2008):

(17) $$\begin{array}{l} Q^{\to }=\frac{1}{l}\underset{i,j\in N}{\sum }\left[a_{ij}-\frac{k_{i}^{in}k_{i}^{out}}{l}\right]\cdot \delta_{m_{i},m_{j},} \end{array}$$

where *l* = ∑_*ij*_*a*_*ij*_ is the number of edges in the graph, and *a*_*ij*_ is defined to be 1 if there is an edge from *j* to *i* and zero otherwise, $k_{i}^{in}$ and $k_{i}^{out}$ are the in- and out-degrees of the node *i*, and δ_*m*_*i*_, *m*_*j*__ is the Kronecker delta, where δ_*m*_*i*_, *m*_*j*__ = 1 if *m_i_* = *m_j_*, and 0 otherwise. High modularity values indicate strong separation of the nodes into modules. *Q* = 0 if nodes are placed at random into modules or if all nodes are in the same cluster (Leicht and Newman, 2008).

To investigate the dynamic changes of modular structures across time, we used normalized mutual information (*nMI*) and normalized variation of information (*nVI*), which measure the similarity between two partitions and the distance in the space of partitions, respectively (cf. Vinh et al., 2010). Thus, these measures show how similar or how different the partitions are. As with the similarity of the network topology indices, we determined these similarity or variation measures for all consecutive community structures and calculated the average in the given similarity or variation matrices.

### Data Reduction and Statistical Analyses

For statistical analyses of *ICI* values, the network vertices of 58 electrode locations oscillating at 10 different frequencies were collapsed into 5 brain sites at each frequency: F (frontal electrodes: Fp1, Fpz, Fp2, F7, … F6, F8), C (central electrodes: FC3, FC1, …, C1, Cz, C2, …, CP2, CP4), P (parieto-occipital electrodes: P7, P5, …, PO8, O1, Oz, O2), LT (left temporal electrodes: FC5, T7, C5, TP7, CP5), and RT (right temporal electrodes: FC6, T8, C6, TP8, CP6). For sake of clarity, we describe here only two experimental conditions: REC and AOT. Results on all four conditions can be found in Supplementary Material. At first, we analyzed the WFC and CFC connectivity strengths (*ICI* values) using a four-way repeated measures ANOVA with a between-subject factor Age and three within-subject factors Condition (REC and AOT), Site (F, C, P, LT, and RsT), and Frequency (10 frequency bins). This analysis was performed separately for WFC and CFC connectivity data determined during the entire 10-s time interval and averaged across eight segments. All other measures that were determined within a 10-s time interval using a sliding time window approach as described above (i.e., mean and standard deviation as well as similarity indices across time and across nodes, complexity measures, and similarity/variation measures of modular organization changes), were analyzed using a two-way repeated measures ANOVA with a between-subject factor Age and a within-subject factor Condition. Greenhouse-Geisser epsilons were used in all ANOVAs for non-sphericity correction when necessary. Fischer's LSD (FLSD) test was employed for *post-hoc* testing. To exclude the influence of costs on the network topology, we also run corresponding ANCOVAs with costs used as covariates. This result is reported in Supplementary Material. To correlate the network topology and network complexity data with perceptual speed (PS) assessed in the three perceptual speed tasks (DSS, DLS, and IP), we calculated composite scores by PCA on the data of these three tasks. Pearson product correlation was then performed with the composite scores of PS and NTD data. To provide further information about the association between WFC and CFC strengths and complexity measures as well as modular organization of the network (e.g., number of modules), we correlated them with each other. It should be indicated here that this was an exploratory study and analyses were performed without adjustment for multiple comparison (cf. Althouse, 2016). We also note here that additional dedicated studies are needed to confirm the results.

## Results

### Age-Related Changes in Network Structure and Network Dynamics

Before describing network topology changes across the lifespan under different conditions, we present the estimation of topology in real and control (i.e., regular and random) networks that were calculated for AOT condition only. This topology estimation is presented in Figure 2. It can be seen that *CC* and correspondingly *E_local_* are greatest in regular or lattice networks and lowest in random networks, whereas *CC* and *E_local_* for the real networks are in between. In contrast, *CPL* is shortest in real networks and longest in lattice networks, while the random networks are in between. Contrary to *CPL*, *E_global_* is lowest in the lattice, highest in random, and lies in between in real networks.

#### Age-Related Changes in WFC and CFC

Statistical analyses of the *ICI* values performed separately for WFC and CFC connectivity data determined during the entire 10-s time interval and averaged across eight 10-s segments as well as similar analyses performed on WFC and CFC strengths determined within single 10-s epoch using sliding time window approach are presented in Supplementary Material and indicate different age-related patterns for WFC and CFC under the four task conditions (see Supplementary Table 1 and Supplementary Figure 1 for details). These analyses showed the same age-related patterns and therefore indicate high generalizability of the data. To prove this statistically, we calculated Cronbach's alpha (α) between mean *ICI* values averaged across eight 10-s segments and strengths determined within a single 10-s time interval. As shown in Supplementary Table 2, both WFC and CFC strength showed high reliabilities (α > 0.895) with exception of CFC strength during REO condition (α = 0.773), which is nevertheless in acceptable range. For the sake of clarity, we further present data for only two task conditions (REC and AOT). To further provide reliability of network topology metrics, we determined these measures for a next 10-s epoch and calculated Cronbach's α between corresponding means determined within two different epochs. As shown in Supplementary Table 3, all topology measures determined for AOT condition showed high reliability (α > 0.900 for network topology means).

**Figure 3:**
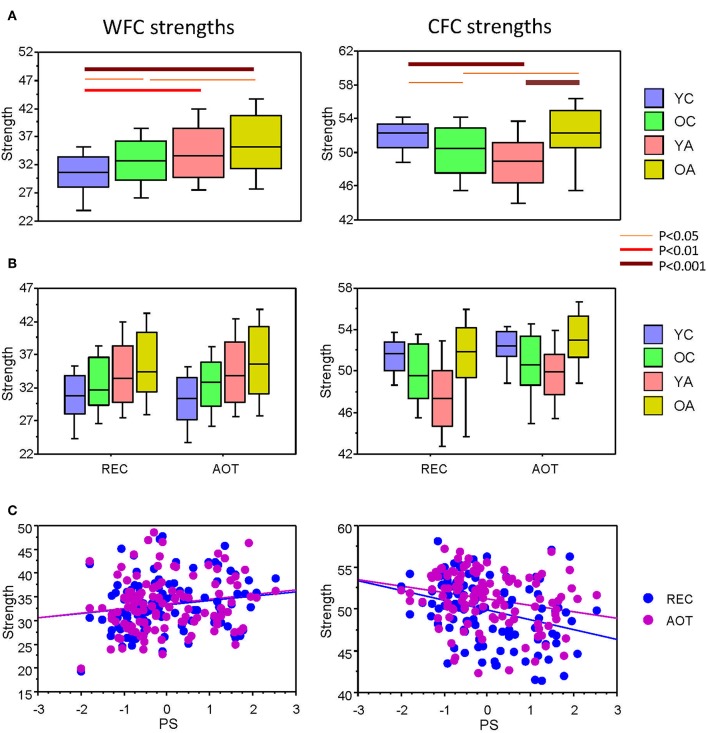
ANOVA results for WFC and CFC strengths and correlation plots of the strengths vs. PS scores. **(A)** Box plots of the WFC (left) and CFC (right) strengths across the lifespan. **(B)** Box plots of the WFC (left) and CFC (right) strengths across the lifespan under the two task conditions (REC and AOT). **(C)** Correlations between strengths (WFC and CFC) and PS composite scores. Age groups: YC, younger children; OC, older children; YA, younger adults; OA, older adults. Conditions: REC, rest with eyes closed; AOT, attended oddball task. WFC, within-frequency coupling; CFC, cross-frequency coupling; PS, perceptual speed.

Here we present CFC and WFC strengths determined within a 10-s time interval to further investigate the influence of WFC and CFC on NTD. As expected and also shown in Supplementary Material for the four task conditions, WFC strength increased practically linearly with age, and CFC strength showed U-shaped relationship across the lifespan (see Figure 3 for details). Statistical analyses of WFC strength revealed only age-related differences [*F*_(3, 107)_ = 5.74, *P* < 0.001, η^2^ = 0.14], whereas CFC strength showed significant effects of both factors Age [*F*_(3, 107)_ = 7.75, *P* < 0.0001, η^2^ = 0.18] and Condition [*F*_(3, 321)_ = 26.25, *P* < 0.0001, η^2^ = 0.20]. There were no significant interactions of these two factors. To exclude the confounding effects of wiring costs, we performed an ANCOVA with costs as covariates. Interestingly, the main effects of the factors Age and Condition were found significant for both WFC [Age: *F*_(3, 105)_ = 7.75, *P* < 0.0001, η^2^ = 0.18; Condition: *F*_(3, 105)_ = 4.07, *P* < 0.05, η^2^ = 0.04] and CFC [Age: *F*_(3, 105)_ = 4.36, *P* < 0.01, η^2^ = 0.11; Condition: *F*_(3, 105)_ = 4.54, *P* < 0.05, η^2^ = 0.04]. To assess the relationship between coupling strengths and cognitive performance, we correlated WFC and CFC strengths with composite scores of the PS (see Methods). As shown in Table 1 and Figure 3C, CFC strength correlated negatively with PS scores, whereas the correlation between WFC strength and PS scores was positive but did not reach the significant level.

**Table 1:** Correlation between PS scores and network variability and complexity measures under the four task conditions.

**Measure**	**REC**		**AOT**	
	***R***	***P***	***R***	***P***
**WFC AND CFC**				
WFC_S	0.168	0.078	0.168	0.078
CFC_S	−0.317	0.0007	−0.237	0.010
***tSD***				
*Sin*	0.363	0.0001	0.548	0.0001
*Sout*	0.227	0.016	0.469	0.0001
*CC*	0.195	0.040	0.090	0.35
*CPL*	0.407	0.0001	0.456	0.0001
*Eloc*	0.413	0.0015	0.333	0.0003
*Eglob*	0.429	0.0001	0.573	0.0001
***nSD***				
*Sin*	0.182	0.056	0.185	0.051
*Sout*	0.160	0.092	0.172	0.072
*CC*	0.251	0.0076	0.181	0.057
*CPL*	0.298	0.0014	0.218	0.022
*Eloc*	0.458	0.0001	0.421	0.0001
*Eglob*	0.154	0.11	0.185	0.052
**COMPLEXITY**				
*GE*	−0.491	0.0001	−0.448	0.0001
*LE*	−0.200	0.035	−0.116	0.23
*Ce*	−0.143	0.14	−0.072	0.45
*Cr*	−0.036	0.71	0.083	0.39
*OdC*	−0.360	0.0001	−0.331	0.0003
*PE*	0.375	0.0001	0.308	0.0009
*CDN*	−0.415	0.0001	−0.409	0.0001
*IDN*	0.306	0.0010	0.237	0.012

*REC, rest with eyes closed; AOT, attended oddball task; WFC, within-frequency coupling; CFC, cross-frequency coupling; PS, perceptual speed; S_in_, in-strength; S_out_, out-strength; CC, clustering coefficient; CPL, characteristic path length; E_local_, local efficiency; E_global_, global efficiency; tSD, standard deviation across time; nSD, standard deviation across nodes; GE, graph energy; LE, Laplacian energy; C_e_, efficiency complexity; C_r_, graph index complexity; OdC, offdiagonal complexity; PE, partition entropy; CDN, correlation dimension of the network; IDN, information dimension of the network*.

#### Mean and Standard Deviation of Network Topology Changes Across the Lifespan

**Figure 4:**
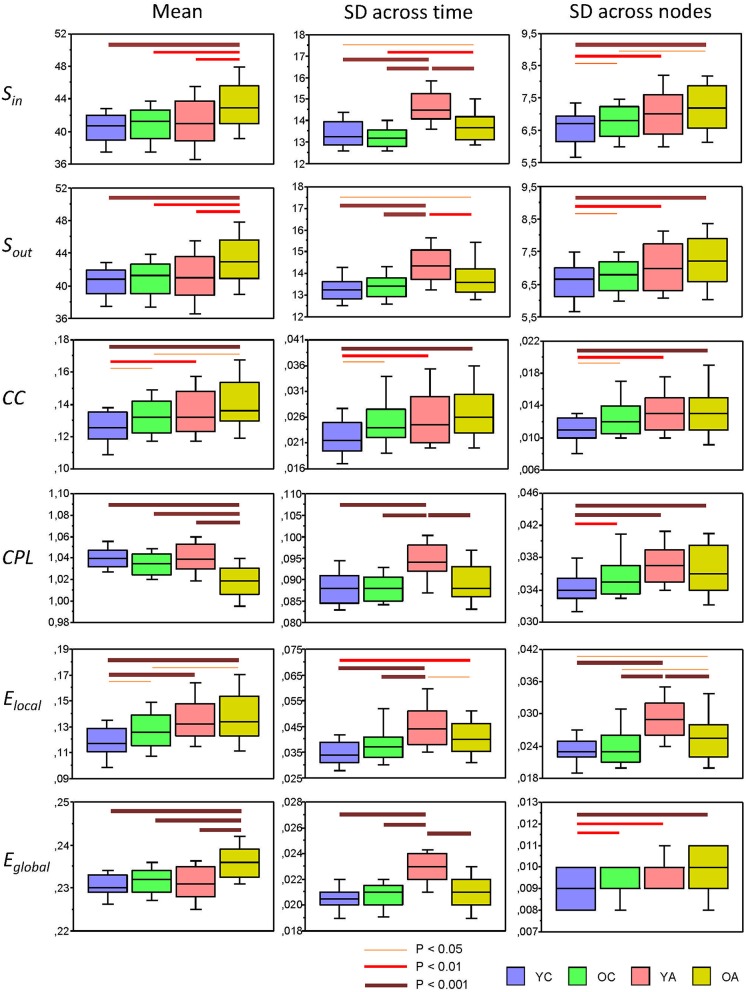
Box plots of the mean and standard deviation (SD) of the six GTA measures across the lifespan. Changes of the mean and SD across the 81 time windows calculated for each network node (*tSD*) and across 580 nodes calculated for each time window (*nSD*) are presented for the six GTA measures: In-Strength (*S_in_*), Out-Strength (*S_out_*), Clustering Coefficient (*CC*), Characteristic Path Length (*CPL*), Local Efficiency (*E_local_*), and Global Efficiency (*E_global_*). For statistical analyses and this presentation, mean and SDs were averaged across nodes (*tSD*) and time windows (*nSD*), respectively. Note that the mean does not depend on averaging procedure. Factor Age is presented here. Differences between age groups were examined by Fisher's LSD *post-hoc* test and are shown by lines' color and thickness.

Using a two-way repeated measures ANOVA with a between-subject factor Age and a within-subject factors Condition, we separately tested the six GTA metrics to show overall (temporal and structural) changes in NTD. Results of these analyses (with exception of the factor Condition, which is outside our focus and was reported previously, cf. Müller et al., 2016) are summarized in Table 2 and Figure 4 (lifespan network topology changes under the four Conditions are shown in Supplementary Figure 2). It can be seen that for all GTA measures both mean and standard deviation vary as function of age. As shown by *post hoc* FLSD test, mean for practically all GTA metrics increases with age and for CPL correspondingly decreases. The dynamic variability (*tSD*) was in most cases highest in YA and for CC in OA, while the structural variability (*nSD*) was highest in YA and OA as compared with children (especially, YC). Besides significant age-related differences between children and adults (see Figure 4 for details), *post hoc* FLSD test revealed also significant differences between YC and OC as well as between YA and OA. In general, YA showed highest *SD* (especially, *tSD*), whereas the magnitude of the GTA metrics (*M*) was greatest in OA. As shown in Supplementary Table 4, ANCOVA controlling for the effects of wiring costs confirmed all main effects of the factor Age shown by ANOVA, which even sometimes were getting stronger.

**Table 2:** ANOVA results for the mean (M) and standard deviation (SD) across time and across nodes for the six GTA measures.

**GTA measures**	**Factors**	***F*-value**	***P*-value**	**Partial eta squared**
**MEAN (M)**				
**S_in_**	Age	*F*_(3, 107)_ = 5.34	***P*** **< 0.005**	η^2^ = 0.13
	Age × Condition	*F*_(3, 107)_ = 1.65	*P* = 0.18	η ^2^ = 0.04
**S_out_**	Age	*F*_(3, 107)_ = 5.32	***P*** **< 0.005**	η^2^ = 0.13
	Age × Condition	*F*_(3, 107)_ = 1.56	*P* = 0.20	η ^2^ = 0.04
*CC*	Age	*F*_(3, 107)_ = 5.49	***P*** **< 0.005**	η^2^ = 0.13
	Age × Condition	*F*_(3, 107)_ = 0.07	*P* = 0.97	η ^2^ = 0.002
*CPL*	Age	*F*_(3, 107)_ = 16.71	***P*** **< 0.0001**	η^2^ = 0.32
	Age × Condition	*F*_(3, 107)_ = 0.83	*P* = 0.48	η ^2^ = 0.02
**E_local_**	Age	*F*_(3, 107)_ = 7.06	***P*** **< 0.0001**	η^2^ = 0.17
	Age × Condition	*F*_(3, 107)_ = 0.19	*P* = 0.90	η ^2^ = 0.01
**E_global_**	Age	*F*_(3, 107)_ = 12.26	***P*** **< 0.0001**	η^2^ = 0.26
	Age × Condition	*F*_(3, 107)_ = 1.34	*P* = 0.27	η ^2^ = 0.04
**STANDARD DEVIATION ACROSS TIME (*****tSD*****)**				
**S_in_**	Age	*F*_(3, 107)_ = 28.04	***P*** **< 0.0001**	η^2^ = 0.44
	Age × Condition	*F*_(3, 107)_ = 2.45	*P* = 0.068	η ^2^ = 0.06
**S_out_**	Age	*F*_(3, 107)_ = 13.30	***P*** **< 0.0001**	η^2^ = 0.26
	Age × Condition	*F*_(3, 107)_ = 2.73	***P*** **< 0. 05**	η ^2^ = 0.07
*CC*	Age	*F*_(3, 107)_ = 4.41	***P*** **< 0.01**	η^2^ = 0.11
	Age × Condition	*F*_(3, 107)_ = 1.78	*P* = 0.16	η ^2^ = 0.05
*CPL*	Age	*F*_(3, 107)_ = 18.67	***P*** **< 0.0001**	η^2^ = 0.34
	Age × Condition	*F*_(3, 107)_ = 1.33	*P* <0.27	η ^2^ = 0.04
**E_local_**	Age	*F*_(3, 107)_ = 9.76	***P*** **< 0.0001**	η^2^ = 0.22
	Age × Condition	*F*_(3, 107)_ = 1.16	*P* <0.33	η ^2^ = 0.03
**E_global_**	Age	*F*_(3, 107)_ = 33.37	***P*** **< 0.0001**	η^2^ = 0.48
	Age × Condition	*F*_(3, 107)_ = 2.15	*P* = 0.10	η ^2^ = 0.06
**STANDARD DEVIATION ACROSS NODES (*****nSD*****)**				
**S_in_**	Age	*F*_(3, 107)_ = 4.25	***P*** **< 0.01**	η^2^ = 0.11
	Age × Condition	*F*_(3, 107)_ = 0.38	*P* = 0.77	η ^2^ = 0.01
**S_out_**	Age	*F*_(3, 107)_ = 3.82	***P*** **< 0.05**	η^2^ = 0.10
	Age × Condition	*F*_(3, 107)_ = 0.64	*P* = 0.59	η ^2^ = 0.02
*CC*	Age	*F*_(3, 107)_ = 4.20	***P*** **< 0.01**	η^2^ = 0.11
	Age × Condition	*F*_(3, 107)_ = 0.13	*P* = 0.94	η ^2^ = 0.004
*CPL*	Age	*F*_(3, 107)_ = 4.85	***P*** **< 0.005**	η^2^ = 0.12
	Age × Condition	*F*_(3, 107)_ = 1.42	*P* = 0.24	η ^2^ = 0.04
**E_local_**	Age	*F*_(3, 107)_ = 13.56	***P*** **< 0.0001**	η^2^ = 0.28
	Age × Condition	*F*_(3, 107)_ = 0.11	*P* = 0.96	η ^2^ = 0.003
**E_global_**	Age	*F*_(3, 107)_ = 4.36	***P*** **< 0.01**	η^2^ = 0.11
	Age × Condition	*F*_(3, 107)_ = 0.40	*P* = 0.75	η ^2^ = 0.01

*S_in_, in-strength; S_out_, out-strength; CC, clustering coefficient; CPL, characteristic path length; E_local_, local efficiency; E_global_, global efficiency. Significant effects (P <0.05) are in bold*.

**Figure 5:**
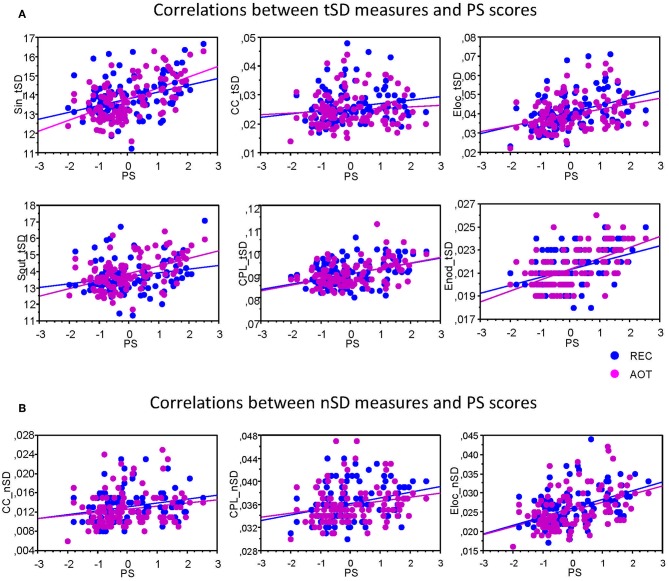
Correlation plots showing Pearson's product correlations between perceptual speed and network variability measures. **(A)** Correlations between temporal variability (*tSD*) and PS composite scores. **(B)** Correlations between nodal variability (*nSD*) and PS composite scores. Pearson's product correlations were calculated for each condition separately (see also Table 1 for details) for each of the six GTA measures: In-Strength (*S_in_*), Out-Strength (*S_out_*), Clustering Coefficient (*CC*), Characteristic Path Length (*CPL*), Local Efficiency (*E_local_*), and Global Efficiency (*E_global_*). Note that significant correlations are presented.

To assess the relationship between temporal and structural network variability and cognitive performance, we correlated *tSD* and *nSD* with composite scores of the PS. Results of these correlations are summarized in Table 1. It can be seen that correlations between *tSD* and perceptual speed scores were significantly positive for practically all NTD data with exception of *CC* (see also Figure 5); *nSD* also correlated positively with PS but to a lesser extent and only for *CC, CPL*, and *E_local_* with some exceptions.

#### Temporal and Nodal Network Similarity Across the Lifespan

**Figure 6:**
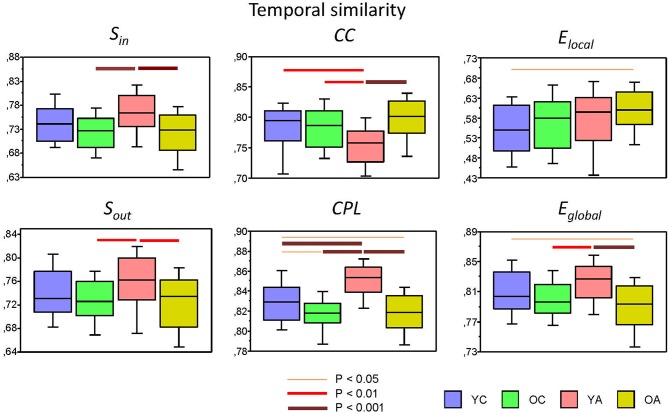
Box plots of the temporal similarity of the six GTA measures across the lifespan. Temporal similarity was calculated by Pearson's product correlation between nodes among the 81 consecutive time windows, resulting in an 81 × 81 symmetric matrix. In this matrix, average strength has been determined as a *global temporal similarity index*. Temporal similarity indices are presented for the six GTA measures across the lifespan (factor Age): In-Strength (*S_in_*), Out-Strength (*S_out_*), Clustering Coefficient (*CC*), Characteristic Path Length (*CPL*), Local Efficiency (*E_local_*), and Global Efficiency (*E_global_*). Differences between age groups were examined by Fisher's LSD *post-hoc* test and are shown by lines' color and thickness.

Results of these analyses for all GTA measures are summarized in Table 3 (with exception of the factor Condition) and Figure 6 (only temporal similarity data, showing strongest lifespan differences, are presented). It can be seen that main effect Age was mostly significant for temporal similarity and to lesser extent for nodal similarity, and only for negative similarity values (with an exception of a significant Age effect in the case of *S_in_* for positive similarity values). The temporal similarity was highest in YA for *S_in_*, *S_out_*, *SPL*, and *E_global_* and lowest for *CC*. The negative nodal similarity was lowest in YA (i.e., the negative similarity was strongest) for *S_in_*, *SPL*, *E_local_*, and *E_global_*, which above all differentiates between YA and OA (YA < OA), indicates thus stronger (negative) similarity in YA. ANCOVA controlling for the effects of wiring costs confirmed all these main effects of the factor Age shown by ANOVA, with some few exceptions (see Supplementary Table 5 for details). Lifespan network topology changes in temporal and nodal similarity under the four task Conditions are shown in Supplementary Figure 3. It can be seen that there were some modulations of lifespan differences by Condition.

**Table 3:** ANOVA results for the temporal and nodal (positive and negative) similarity for the six GTA measures.

**GTA measures**	**Factors**	***F*-value**	***P*-value**	**Partial eta squared**
**TEMPORAL NETWORK SIMILARITY**				
**S_in_**	Age	*F*_(3, 107)_ = 6.36	***P*** **< 0.001**	η^2^ = 0.15
	Age × Condition	*F*_(3, 107)_ = 2.74	***P*** **< 0.05**	η ^2^ = 0.07
**S_out_**	Age	*F*_(3, 107)_ = 3.70	***P*** **< 0.05**	η^2^ = 0.09
	Age × Condition	*F*_(3, 107)_ = 1.39	*P* = 0.25	η ^2^ = 0.04
*CC*	Age	*F*_(3, 107)_ = 5.96	***P*** **< 0.001**	η^2^ = 0.14
	Age × Condition	*F*_(3, 107)_ = 3.40	***P*** **< 0.05**	η ^2^ = 0.09
*CPL*	Age	*F*_(3, 107)_ = 23.18	***P*** **< 0.0001**	η^2^ = 0.39
	Age × Condition	*F*_(3, 107)_ = 3.32	***P*** **< 0.05**	η ^2^ = 0.09
**E_local_**	Age	*F*_(3, 107)_ = 1.92	*P* = 0.13	η^2^ = 0.05
	Age × Condition	*F*_(3, 107)_ = 0.49	*P* = 0.69	η ^2^ = 0.01
**E_global_**	Age	*F*_(3, 107)_ = 7.29	***P*** **< 0.0001**	η^2^ = 0.17
	Age × Condition	*F*_(3, 107)_ = 0.13	*P* = 0.94	η ^2^ = 0.004
**NETWORK SIMILARITY ACROSS NODES (POSITIVE)**				
**S_in_**	Age	*F*_(3, 107)_ = 2.90	***P*** **< 0.05**	η^2^ = 0.08
	Age × Condition	*F*_(3, 107)_ = 0.22	*P* = 0.88	η ^2^ = 0.01
**S_out_**	Age	*F*_(3, 107)_ = 2.54	*P* = 0.061	η^2^ = 0.07
	Age × Condition	*F*_(3, 107)_ = 0.06	*P* = 0.98	η ^2^ = 0.002
*CC*	Age	*F*_(3, 107)_ = 2.29	*P* = 0.082	η^2^ = 0.06
	Age × Condition	*F*_(3, 107)_ = 0.14	*P* = 0.94	η ^2^ = 0.004
*CPL*	Age	*F*_(3, 107)_ = 1.12	*P* = 0. 34	η^2^ = 0.03
	Age × Condition	*F*_(3, 107)_ = 0.79	*P* = 0.50	η ^2^ = 0.02
**E_local_**	Age	*F*_(3, 107)_ = 1.31	*P* = 0.34	η^2^ = 0.03
	Age × Condition	*F*_(3, 107)_ = 0.32	*P* = 0.81	η ^2^ = 0.01
**E_global_**	Age	*F*_(3, 107)_ = 2.02	*P* = 0.12	η^2^ = 0.05
	Age × Condition	*F*_(3, 107)_ = 0.34	*P* = 0.80	η ^2^ = 0.01
**NETWORK SIMILARITY ACROSS NODES (NEGATIVE)**				
**S_in_**	Age	*F*_(3, 107)_ = 2.90	***P*** **< 0.05**	η^2^ = 0.08
	Age × Condition	*F*_(3, 107)_ = 0.22	*P* = 0.88	η ^2^ = 0.01
**S_out_**	Age	*F*_(3, 107)_ = 1.23	*P* = 0.30	η^2^ = 0.03
	Age × Condition	*F*_(3, 107)_ = 0.47	*P* = 0.70	η ^2^ = 0.01
*CC*	Age	*F*_(3, 107)_ = 0.25	*P* = 0.86	η^2^ = 0.01
	Age × Condition	*F*_(3, 107)_ = 0.83	*P* = 0.48	η ^2^ = 0.02
*CPL*	Age	*F*_(3, 107)_ = 5.67	***P*** **< 0.001**	η^2^ = 0.14
	Age × Condition	*F*_(3, 107)_ = 0.22	*P* = 0.88	η ^2^ = 0.01
**E_local_**	Age	*F*_(3, 107)_ = 3.66	***P*** **< 0.05**	η^2^ = 0.09
	Age × Condition	*F*_(3, 107)_ = 1.52	P = 0.21	η ^2^ = 0.04
**E_global_**	Age	*F*_(3, 107)_ = 8.38	***P*** **< 0.0001**	η^2^ = 0.19
	Age × Condition	*F*_(3, 107)_ = 0.80	*P* = 0.50	η ^2^ = 0.02

*S_in_, in-strength; S_out_, out-strength; CC, clustering coefficient; CPL, characteristic path length; E_local_, local efficiency; E_global_, global efficiency. Significant effects (P <0.05) are in bold*.

### Age-Related Changes in Network Complexity

**Figure 7:**
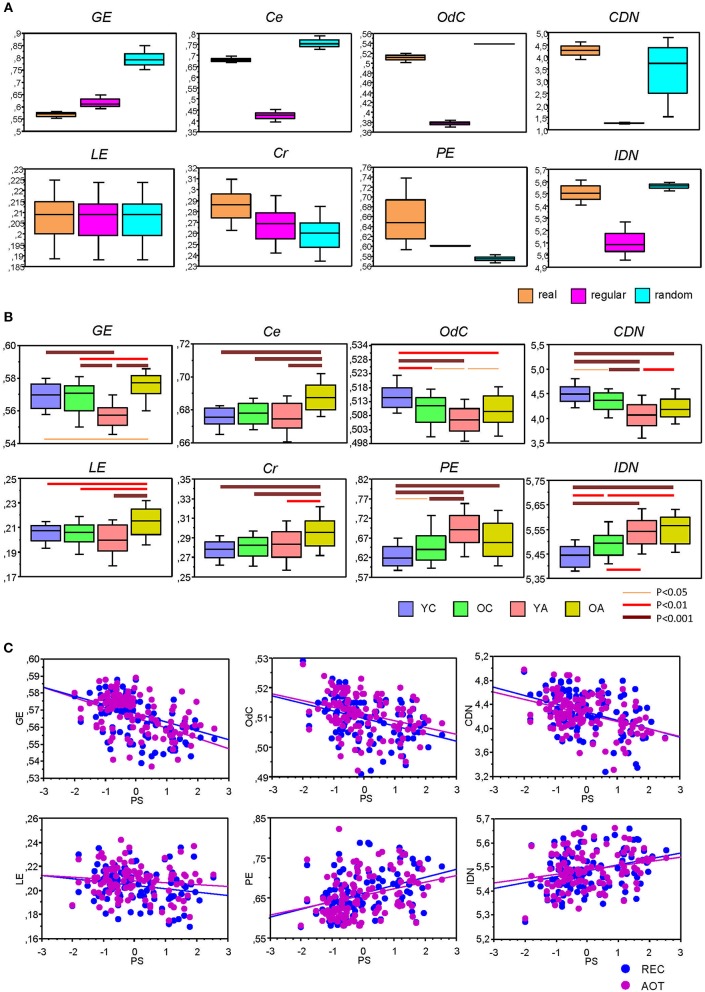
Box plots of the network complexity measures for the real and control networks (regular and random) as well as for the lifespan-related changes. **(A)** Box plots of the eight complexity measures for real, regular, and random networks. For this presentation, data were determined for AOT condition and averaged across all subjects. **(B)** Box plots of the network complexity changes across the lifespan. Differences between age groups were examined by Fisher's LSD *post-hoc* test and are shown by lines' color and thickness. **(C)** Correlation plots of Pearson's product correlations between perceptual speed and network complexity measures. Pearson's product correlations showing significant relationships are presented (see also Table 5 for details). Age groups: YC, younger children; OC, older children; YA, younger adults; OA, older adults. Conditions: REC, rest with eyes closed; AOT, attended oddball task. Complexity measures: *GE*, graph energy; *LE*, Laplacian energy; *C_e_*, efficiency complexity; *C_r_*, graph index complexity; *OdC*, offdiagonal complexity; *PE*, partition entropy; *CDN*, correlation dimension of the network; *IDN*, information dimension of the network. PS, perceptual speed scores.

Before describing network complexity changes across the lifespan under different conditions, we compared complexity in real and control (i.e., regular and random) networks. Normally, complexity is highest in random networks and lowest in regular or lattice networks, while the real networks were expected to lie in between. This expectation was mostly true with several exceptions, especially with regard to energy-based measures. As shown in Figure 7A, *GE* was lowest in real networks and highest in random networks, while *LE* was contrary slightly higher in real networks and equal in regular and random networks. *C_r_* and *PE* were highest in real networks and lowest in random networks. It should be noted here that *PE* is dependent on partition or number of modules (*r* = 0.990, *p* < 0.0001), and therefore, this result should be treated with caution, especially with regard to random networks because of amorphous or random modularity structure. As shown, *IDN* as well as *OdC* and *C_e_* were highest in random networks and lowest in regular networks, while they were in between for the real networks. Finally, *CDN* was highest in real networks, lowest in regular networks, and was in between for random networks.

Lifespan differences in network complexity under the four experimental conditions were tested using a two-way repeated measures ANOVA with a between-subject factor Age and a within-subject factor Condition. Results of these analyses are presented in Table 4 and Figure 7B. It can be seen that main effects of the factor Age was highly significant for all complexity measures. These effects were also confirmed by ANCOVA, excluding the confounding effects of wiring costs (see Supplementary Table 6 for details). The energy measures *GE* and *LE* were greatest in OA and lowest in YA. The product measures *C_r_* and *C_e_* were also greatest in OA as compared to all other age groups. *OdC* and *CDN* showed U-shaped relationship across the lifespan and were greatest in YC and lowest in YA. Contrary, *PE* and *IDN* showed inverted U-shaped relationship and continuous increase across the lifespan, respectively.

**Table 4:** ANOVA results for the network complexity and modular organization measures.

**Measures**	**Factors**	***F*-value**	***P*-value**	**Partial eta squared**
**COMPLEXITY MEASURES**				
*GE*	Age	*F*_(3, 107)_ = 16.69	***P*** **< 0.0001**	η^2^ = 0.32
	Age × Condition	*F*_(3, 107)_ = 2.78	***P*** **< 0.05**	η^2^ = 0.07
*LE*	Age	*F*_(3, 107)_ = 7.76	***P*** **< 0.0001**	η^2^ = 0.18
	Age × Condition	*F*_(3, 107)_ = 2.07	*P* = 0.11	η^2^ = 0.06
**C_e_**	Age	*F*_(3, 107)_ = 13.67	***P*** **< 0.0001**	η^2^ = 0.28
	Age × Condition	*F*_(3, 107)_ = 1.13	*P* = 0.34	η^2^ = 0.03
**C_r_**	Age	*F*_(3, 107)_ = 7.28	***P*** **< 0.0001**	η^2^ = 0.17
	Age × Condition	*F*_(3, 107)_ = 1.57	*P* = 0.20	η^2^ = 0.04
*OdC*	Age	*F*_(3, 107)_ = 8.83	***P*** **< 0.0001**	η^2^ = 0.20
	Age × Condition	*F*_(3, 107)_ = 0.63	*P* = 0.60	η^2^ = 0.02
*PE*	Age	*F*_(3, 107)_ = 10.41	***P*** **< 0.0001**	η^2^ = 0.23
	Age × Condition	*F*_(3, 107)_ = 1.51	*P* = 0.22	η^2^ = 0.04
*CDN*	Age	*F*_(3, 107)_ = 16.67	***P*** **< 0.0001**	η^2^ = 0.32
	Age × Condition	*F*_(3, 107)_ = 0.84	*P* = 0.47	η^2^ = 0.02
*IDN*	Age	*F*_(3, 107)_ = 17.40	***P*** **< 0.0001**	η^2^ = 0.33
	Age × Condition	*F*_(3, 107)_ = 0.89	*P* = 0.45	η^2^ = 0.02
**MODULAR ORGANIZATION MEASURES**				
*Q*	Age	*F*_(3, 107)_ = 4.03	***P*** **< 0.01**	η^2^ = 0.10
	Age × Condition	*F*_(3, 107)_ = 1.12	*P* = 0.35	η^2^ = 0.03
*NoFM*	Age	*F*_(3, 107)_ = 11.31	***P*** **< 0.0001**	η^2^ = 0.24
	Age × Condition	*F*_(3, 107)_ = 1.51	*P* = 0.22	η^2^ = 0.04
*nMI*	Age	*F*_(3, 107)_ = 16.81	***P*** **< 0.0001**	η^2^ = 0.32
	Age × Condition	*F*_(3, 107)_ = 0.52	*P* = 0.67	η^2^ = 0.01
*nVI*	Age	*F*_(3, 107)_ = 5.0	***P*** **< 0.005**	η^2^ = 0.12
	Age × Condition	*F*_(3, 107)_ = 0.82	*P* = 0.49	η^2^ = 0.02

*GE, graph energy; LE, Laplacian energy; C_e_, efficiency complexity; C_r_, graph index complexity; OdC, offdiagonal complexity; PE, partition entropy; CDN, correlation dimension of the network; IDN, information dimension of the network; Q, modularity; NofM, number of modules; nMI = normalized mutual information; nVI = normalized variation of information. Significant effects (P <0.05) are in bold*.

To assess the relationship between network complexity and cognitive performance (PS), we correlated network complexity measures with the composite scores of the PS (see Methods). Results of these correlations are summarized in Table 1 and Figure 7C. It can be seen that most of the complexity measures (i.e., *GE, LE, OdC*, and *CDN*) correlated significant negatively with PS scores; *PE* and *IDN* correlated significant positively, and two product complexity measures (i.e., *C_e_* and *C_r_*) did not show significant correlations.

Further, to investigate the influence of WFC and CFC on network complexity, we correlated them with the complexity measures. Correlation of WFC and CFC strengths with complexity measures are summarized in Table 5 and Supplementary Figure 4. All correlations were statistically significant, whereby most of them were positive and only some of them were negative: WFC strength correlated negatively with *GE, OdC*, and *CDN*, while CFC strength correlated negatively with *PE* and *IDN*. It should be noted that the former three complexity measures (i.e., *GE, OdC* and *CDN*) showed a U-shaped relationship across the lifespan, and the last two measures (*PE* and *IDN*)—an inverted U-shaped and linear-positive, respectively. Thus, lifespan-related changes in network complexity seem to be predictable for the sign of correlation in this case.

**Table 5:** Correlation between WFC and CFC strengths and complexity measures.

**Measure**	**REC**		**AOT**	
	***R***	***P***	***R***	***P***
**WFC**				
*GE*	−0.307	0.001	−0.268	0.0044
*LE*	0.578	0.0001	0.657	0.0001
*Ce*	0.736	0.0001	0.789	0.0001
*Cr*	0.810	0.0001	0.855	0.0001
*OdC*	−0.744	0.0001	−0.798	0.0001
*PE*	0.701	0.0001	0.754	0.0001
*CDN*	−0.616	0.0001	−0.594	0.0001
*IDN*	0.778	0.0001	0.768	0.0001
**CFC**				
*GE*	0.727	0.0001	0.706	0.0001
*LE*	0.768	0.0001	0.667	0.0001
*Ce*	0.568	0.0001	0.463	0.0001
*Cr*	0.492	0.0001	0.391	0.0001
*OdC*	0.680	0.0001	0.639	0.0001
*PE*	−0.579	0.0001	−0.543	0.0001
*CDN*	0.398	0.0001	0.370	0.0001
*IDN*	−0.343	0.0002	−0.269	0.0042

*GE, graph energy; LE, Laplacian energy; C_e_, efficiency complexity; C_r_, graph index complexity; OdC, offdiagonal complexity; PE, partition entropy; CDN, correlation dimension of the network; IDN, information dimension of the network*.

### Dynamic Changes of Modular Organization of the Networks Across the Lifespan

Modular organization of the network was captured above by the *PE*, demonstrating an inverted U-shaped relationship across the lifespan. We showed that this entropy or complexity measure was dependent on the number of modules. Here, we investigated modular organization of the networks and its dynamic changes or temporal similarity of community structures, i.e., we are aiming to show how stable or unstable the community structures are across time. For these purposes, we used modularity analyses to partition the HFNs at each time window, and then calculated normalized mutual information (*nMI*) and variance information (*nVI*) between consecutive community structures to each other. For statistical analyses, we calculated the average in the given similarity or variation matrices.

**Figure 8:**
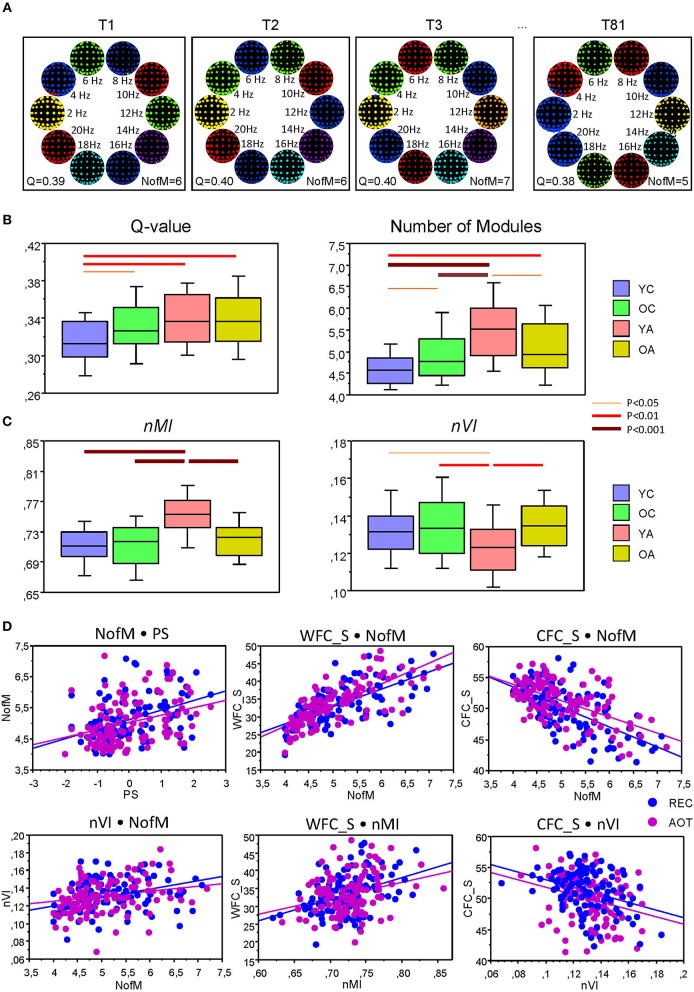
Modular organization of the HFNs and its changes across the lifespan. **(A)** Modular organization of the HFNs displayed for four exemplary networks or time windows. Module affiliation is indicated by color. Note that community structures are organized according to the principle of frequency allocation and frequency interaction, i.e., nodes or electrodes that share the same oscillation frequency or stay at a simple ratio (e.g., 1:2 or 1:4) mostly belong to the same community. **(B)** Changes of Q-values and number of modules (NofM) across the lifespan. **(C)** Temporal changes of similarity and variation of modular organization measured by *nMI* and *nVI* across the lifespan. **(D)** Correlation plots for the correlations between number of modules (NofM) and PS scores as well as correlations of WFC and CFC strengths with *nMI* and *nVI* indices.

Modular organization of the HFNs is displayed in Figure 8A for four exemplary networks or time windows. It can be seen that the modularity or community structures are organized according to the principle of frequency allocation and frequency interaction, i.e., nodes or electrodes that share the same oscillation frequency or stay at a simple ratio (e.g., 1:2 or 1:4) mostly belong to the same community structure. The partition of HFNs was non-random and slightly but significantly worse in YC, as indicated by Q-value (see Table 4 and Figure 8B for details). YC showed also in total a lowest number of modules, which was highest in YA, apparently through better separation of the frequencies. A two-way repeated measures ANOVA for *nMI* and *nVI* measures revealed significant main effect of the factor Age, which was also confirmed by ANCOVA, excluding the confounding effects of wiring costs (see Table 4 and Supplementary Table 6 for details). As shown in Figure 8C, *nMI* was highest and *nVI* lowest in YA as compared with all other age groups, indicating stronger similarity of community structures in YA across time.

Further, to investigate the influence of WFC and CFC as well as that of cognitive abilities of the subjects on modularity structure, we correlated them with the number of modules, *nMI* and *nVI* measures. Results of these correlation analyses are summarized in Table 6 and Figure 8D. PS scores correlated significant positively with the number of modules and with the *nMI* but significant negatively with *nVI*. Interestingly, WFC strength correlated significant positively with *nMI*, and CFC strength correlated significant negatively with *nVI*. Thus, WFC strength determines the similarity of community structures, while CFC strength causes the (reduced) variation of community structures. Similarly, WFC strength correlated significant positively and CFC strength significant negatively with the *NofM*. Interestingly, both *nMI* and *nVI* correlated positively with *NofM*, i.e., high number of modules is associated with high similarity and at the same time with high variability of modular structure of the networks (see Table 6 and Figure 8D for details).

**Table 6:** Correlation between WFC and CFC strengths, indices of modular organization, and perceptual speed.

**Measure**	**REC**		**AOT**	
	***R***	***P***	***R***	***P***
NofM × PS	0.418	0.0001	0.331	0.0004
NofM × WFC_S	0.667	0.0001	0.743	0.0001
NofM × CFC_S	−0.636	0.0001	−0.575	0.0001
NofM × nMI	0.296	0.0015	0.177	0.062
NofM × nVI	0.245	0.0094	0.367	0.0001
nMI × PS	0.374	0.0001	0.443	0.0001
nVI × PS	−0.179	0.060	−0.250	0.0079
nMI × WFC_S	0.410	0.0001	280	0.0028
nMI × CFC_S	−0.032	0.73	0.052	0.59
nVI × WFC_S	−0.018	0.85	0.142	0.14
nVI × CFC_S	−0.278	0.0030	−0.344	0.0001

*NofM, number of modules; PS, perceptual speed; WFC_S, within-frequency coupling strength; CFC_S, cross-frequency coupling strength; nMI, normalized mutual information; nVI = normalized variation of information*.

## Discussion

We examined network structure and network dynamics during rest and auditory oddball performance across the lifespan. For this examination, we constructed hyper-frequency networks based on WFC and CFC, and explored structural and dynamic changes of HFNs. The main findings are that: (a) WFC increased linearly across the lifespan, whereas CFC decreased from YC to YA and increased again in OA; (b) the magnitude of GTA measures increased rather linearly with age (with exception of *CPL*, which correspondingly was decreasing), while *SD* demonstrates inverse U-shaped relationship with greatest *SD* in YA, at least when calculated across time; (c) temporal and to some extend structural or nodal similarity of network topology (mostly with respect to negative correlation values) seems to coincide with SD changes, i.e., stronger variability (*SD*) is related to higher similarity between consecutive time windows or nodes; (d) complexity measures showed different lifespan-related patterns including U-shaped relationship for *GE, LE, OdC*, and *CDN*, inverted U-shaped relationship for *PE*, and linear-like relationship for *C_e_*, *C_r_*, and *IDN* measures; (e) modular organization of the networks is characterized by higher number of modules and stronger similarity of community structures across time in YA; (f) number of modules as well as PS scores correlated positively with WFC strength and negatively with CFC strength.

The fact that WFC increased continuously with age was not unexpected (cf. Müller et al., 2009), while the U-shaped relationship of the CFC across the lifespan with the lowest CFC in YA was somewhat surprising. However, when taking into account the suggestion mentioned in the introduction that CFC might be seen as one of the mechanisms underlying neural communication *between* different cell assemblies, the decrease of CFC from YC to YA and its subsequent increase in OA become clear. The negative relationship between CFC strength and the number of modules (and also the PS scores) just strengthen our suggestion. It seems that a good separation between cell assemblies can be guaranteed when the connectivity between cell assemblies, which is mostly provided by CFC, is reduced. In OA, both WFC and CFC increase, and they have lower number of modules than YA. Apparently, increase in CFC in OA (and also in children) leads to confusion of separated cell assemblies to bigger ones indicated also by a smaller number of modules as compared with YA. Moreover, this confusion of cell assemblies seems to reduce the cognitive ability in perceptual speed. This observation is in line with the so-called differentiation hypothesis during development (Garrett, 1946) and dedifferentiation hypothesis of cognitive aging (Baltes et al., 1980; Baltes and Lindenberger, 1997; Hülür et al., 2015). Interestingly, *CC* increased and *CPL* was going shorter with age, whereby both *E_local_* and *E_global_* correspondingly increased. Notably, *CPL* and also *E_global_* did not differ in children and YA. YA and OA did not differ in *CC* and *E_local_* but showed strong (significant) differences in *CPL* and *E_global_*. On the one hand, high CC and shorter CPL (as well as high *E_local_* and *E_global_*) are signs of strong segregation and integration of neural processes and point out that the networks under investigation are small-world networks (Watts and Strogatz, 1998; Achard and Bullmore, 2007). On the other hand, stronger integration of neuronal elements or processes (shorter *CPL* and higher *E_global_*) in OA as compared to YA is apparently related to higher CFC and presumably indicates loss of independence of separate cell assemblies, although high *CC* and *E_local_* indicate preservation of high degree in *local* segregation processes. It seems that the organization of neural networks in OA moves toward a more consolidated structure (shorter *CPL* and higher *E_global_*) with higher local (*CC* and *E_local_*) but not global (lower number of modules) separability (cf. Baltes and Lindenberger, 1997; Ghisletta and Lindenberger, 2003), which are accompanied by increased WFC and CFC in OA as compared with YA and children. The positive correlation between number of modules and PS indicates that this reduction of number of modules is not functional, i.e., do not promote cognitive or perceptual speed performance.

Further, we found higher temporal (*tSD*) and nodal (*nSD*) variability in adults as compared with children, and especially in YA and for temporal variability. As mentioned, transient temporal fluctuations in brain signal or brain signal variability are mostly related to the high cognitive performance and mental activity, and provide important information about network dynamics and brain states (Deco et al., 2011; Garrett et al., 2011, 2015; McIntosh et al., 2014; Sleimen-Malkoun et al., 2015). This relationship is strengthened by positive correlation between network variability and PS. In the present study, we calculated variability of the *network topology* instead of signal variability, which seems to be an important marker of development and aging. In contrast to brain signal variability, NTD variability represent a high order of varying brain processes, including not only variability in a signal but rather variability of networks or network topologies capturing all the complex interactions between different signals or network components. Since we used HFNs in the present study, this variability (primarily) encompasses interactions within and between different frequencies. Calculation of structural or nodal variability, which also correlated positively with PS scores, although to a lesser extent than temporal variability, extends our understanding about the nature of variable states of the brain. Presumably, this variability measure, which showed predominantly significant differences between adults and children, and especially between YC and OC, seems to be an effective indicator for *differentiation* processes in the brain. Furthermore, it should be noted that nodal variability of *E_local_* did not show significant differences between YC and OC but showed strong significant differences between YA and OA (and also children) that might be also a sign of *dedifferentiation*. *E_local_* is a measure of the segregation of a network and indicates efficiency of information transfer in the immediate neighborhood of a node (Achard and Bullmore, 2007), therefore, decreased nodal variability of *E_local_* apparently indicates a loss in plasticity of local processes, although the local information transfer in OA remains strong (high magnitude of *E_local_*).

Interestingly, high NTD variability in YA was also accompanied by high NTD similarity, at least by high temporal NTD similarity, with exception of *CC* and *E_local_* topology measures, which are responsible for local processes. High temporal NTD similarity indicates that although the topology of each node in the network strongly varies across time, the network structure itself with regards to these measures (i.e., *S_in_*, *S_out_*, *SPL*, and *E_global_*) remains more or less stable allowing transportation of relevant information from one variable state to the other variable state and provides HFN self-similarity.

As indicated above, network complexity measures, which provide further important information about the geometry or structure of complex networks beyond purely topological aspects, showed different lifespan-related patterns including U-shaped, inverted U-shaped, and linear-like relationships. Graph energy measures (*GE* and *LE*) indicated U-shaped relationship with lowest network energy in YA and highest energy in OA. Graph-energy concept has a chemical motivation and is related to the total energy of π-electron orbits (Gutman and Zhou, 2006; Zhou et al., 2007; Kim and Wilhelm, 2008). As shown in our simulation results when comparing real and control networks, *GE* is smallest in real networks and highest in random networks, whereas *LE* is relatively similar in real and control networks. Since energy of a graph is represented on the sum of absolute eigenvalues, it can provide information about capacity or connectedness of the network. Children and especially OA seem to have high capacity or connectedness of the networks but energy (*GE* and *LE*) correlated negatively with cognitive performance (PS scores). Presumably, these hyperenergetic network states reduce the cognitive control or produce noise in the system with more stochastic than deterministic components (cf. McIntosh et al., 2010; Müller and Lindenberger, 2012).

The product complexity measures (*C_e_* and *C_r_*) increased monotonously with age, although *C_e_* does not differ in YA and children. Comparison of real networks with control networks showed that *C_e_* was lowest in regular and highest in random networks with real networks lying in between, whereby *C_r_* was highest in real networks and lowest in random networks. Interestingly, both measures, which were highest in OA, did not correlate with PS scores. As noted in Kim and Wilhelm (2008), *C_e_* can be maximal for graphs with a medium number of links, but nevertheless quite high efficiency. OA showed high level of local and global efficiency and correspondingly high efficiency complexity. The graph index complexity *C_r_* is based on the calculation of the largest eigenvalue and is apparently related to the high connectedness and interlacement. So, both product complexity measures point at strong connectedness but also at high efficiency in OA. Nevertheless, it remains to be seen whether this higher complexity calculated by these two measures reflects the optimal structure and functionality of the networks.

The two entropy measures (i.e., *OdC* and *PE*) quantifying the diversity of different topological features showed U-shaped and inverted U-shaped relationships, respectively. While *OdC* correlated significant negatively with PS scores, *PE* correlated significant positively with PS scores. When compared with control networks, *OdC* was lowest in regular networks and strongest in random networks, while *PE* was highest in real networks and lowest in random networks. It should be noted here that *PE* was strongly related to the number of modules derived by the network partition and can be biased by the number of modules. As noted in our previous work (Müller et al., 2016), HFNs are small-world networks characterized by a topology with a slight tendency to random characteristics and are organized in such a way that if there is WFC only, such a network will be akin to a regular network, and increasing CFC will increase its randomness. A small-world network would represent a balance between WFC and CFC. In the case of *OdC*, the entropy is calculated across off-diagonals, whereby first of them include mostly WFC and further include CFC only. These peculiarities in the calculation procedure are presumably reflected in the correlations between *OdC* on one side, and WFC and CFC strengths on the other. The fact that *OdC* correlated significant negatively with WFC and significant positively with CFC indicates that high WFC supports regularity and high CFC is responsible for randomness of the network. This association of *OdC* with WFC and CFC apparently designates the lifespan-related differences, i.e., the U-shaped relationship across the lifespan.

Like the *OdC* and the energy measures, the dimensionality measure *CDN* revealed a U-shaped lifespan-related relationship and correlated significant negatively with PS scores, while *IDN* increased practically linearly with age, and correlated significant positively with PS scores. When compared with control networks, both *CDN* and *IDN* were lowest in regular networks but *CDN* was strongest in real networks and *IDN* was strongest in random networks. Moreover, *CDN* correlated significant negatively with WFC strength and significant positively with CFC strength, while *IDN* showed inverse relationship. This is apparently the reason that YA, showing relatively high WFC but lowest CFC strengths, are characterized by low *CDN* and high *IDN*. The significant correlation with PS scores (negative in the case of the *CDN* and positive in the case of the *IDN*) underlines the functional diversity of these network complexity or dimensionality measures with regard to cognitive performance. In general, the relations or differences between the WFC and CFC strengths seem to be dispositive for the network topology and network architecture, as well as for its complexity and functionality. Further studies are necessary for better understanding of natural and systemic diversity of HFNs and complex interplay of network components and underlying neural processes. Furthermore, because of the fact that we used an absolute threshold for network construction, which can constitute a bias for different groups, a proportional threshold could be used in further analyses in order to increase the robustness of results. To control for the bias mentioned before and to exclude the confounding effects of wiring costs, we performed in this study an ANCOVA with costs as covariates. It has been shown that the confounding effects of costs on lifespan differences was inconsiderable. Nevertheless, the use of proportional thresholds or other threshold selection strategies could provide further important information about NTD with regard to different ages.

In sum, the present work showed that HFNs can be characterized by a battery of metrics, designed to systematically quantify the cross-spectral relationships constructed by WFC and CFC. The HFNs possess small-world network topology and lead to different lifespan-related patterns with regard to network variability and network complexity dynamics. This dynamics is characterized by mostly inverse U-shaped temporal network variability and similarity across the lifespan, and different lifespan-related patterns in network complexity, which depend on the WFC-CFC balance in the network. Importantly, all these dynamic changes are mostly related to cognitive performance of the subjects and represent economically plausible features (i.e., high local and global efficiency of parallel information processing on the basis of low network connection costs) at practically all frequencies investigated in the study, in line with other biological and social systems. Future research would clarify how different patterns in network topology, variability and complexity dynamics contribute to functional activity and reactivity of brain processes and stimulate or boost development and aging. Furthermore, fine-tuned balance between WFC and CFC in NTD and network complexity underlying behavior can be an important factor in various forms of psychopathology. The clinical and diagnostic utility of network topology and network complexity measures in these and related contexts remains to be explored.

## Author Contributions

VM, TvO, and UL designed the study. VM acquired and analyzed the data. VM, DP, TvO, RS-M, VJ, and UL discussed the results and wrote the article. All authors read and approved the final version of the manuscript.

### Conflict of Interest Statement

The authors declare that the research was conducted in the absence of any commercial or financial relationships that could be construed as a potential conflict of interest.
