# Supplementary material for: Lifespan Changes in Network Structure and Network Topology Dynamics During Rest and Auditory Oddball Performance
Source: Front Aging Neurosci. 2019 Jun 11;11:138. doi: 10.3389/fnagi.2019.00138 (PMC6580332; doi:10.3389/fnagi.2019.00138)
Supplement: Supplementary file 1 [file Data_Sheet_1.PDF]

frontiers

*Supplementary Material*

# Lifespan Changes in Network Structure and Network Topology Dynamics during Rest and Auditory Oddball Performance

**Viktor Müller*, Viktor Jirsa, Dionysios Perdikis, Rita Sleimen-Malkoun, Timo von Oertzen, and Ulman Lindenberger**

***Correspondence:** Dr. Viktor Müller, Email: vmueller@mpib-berlin.mpg.de

## 1 Age-related changes in WFC and CFC

Statistical analyses of WFC and CFC of the *ICI* data using a two-way repeated measures ANOVA with a between-subject factor Age (YC, OC, YA, and OA) and a within-subject factors Condition (REC, REO, UOT, and AOT) revealed a significant main effect of Age for both WFC and CFC; whereas main effect of Condition and interaction of factors Age and Condition were significant only for CFC (see Supplementary Table 1 and Fig. 1 for details). The post-hoc Fischer's LSD test for WFC-values showed significantly lower coupling in YC than in other age groups (YC < OC, $P < 0.05$; YC < YA, $P < 0.01$; YC < OA, $P < 0.0001$), while CFC-values were significantly lower in YA than in other age groups (YA < OC, $P < 0.005$; YA < YC, $P < 0.0001$; YA < OA, $P < 0.0001$). The WFC and CFC strength determined within a 10-s epoch using a sliding time window approach showed very similar results (see Supplementary Table 1 and Fig. 1 for details), with an exception that post-hoc tests showed in addition significant differences between OC and OA for both WFC and CFC strengths ($P < 0.05$), whereby the differences between OC and YA were only approximately significant. In accordance with these similarities, Cronbach's alpha test showed high consistency for mean *ICI* values averaged across eight 10-s segments and strengths determined within a 10-s time interval using a sliding time window approach (see Supplementary Table 2 for details).

**Supplementary Table 1.** ANOVA results for the mean *ICI* values and coupling strengths determined separately for within- and cross-frequency coupling

| Factors | F-value | P-value | Partial eta squared |
|---|---|---|---|
| | Mean *ICI* (WFC) | | |
| Age | $F_{3,107} = 5.71$ | **P < 0.005** | $\eta^2 = 0.14$ |
| Condition | $F_{3,321} = 0.46$ | P = 0.68 | $\eta^2 = 0.004$ |
| Age × Condition | $F_{9,321} = 0.97$ | P = 0.46 | $\eta^2 = 0.03$ |
| | Mean *ICI* (CFC) | | |
| Age | $F_{3,107} = 11.29$ | **P < 0.0001** | $\eta^2 = 0.24$ |
| Condition | $F_{3,321} = 91.41$ | **P < 0.0001** | $\eta^2 = 0.46$ |
| Age × Condition | $F_{9,321} = 6.87$ | **P < 0.0001** | $\eta^2 = 0.16$ |
| | Strength (WFC) | | |
| Age | $F_{3,107} = 6.08$ | **P < 0.005** | $\eta^2 = 0.15$ |
| Condition | $F_{3,321} = 2.63$ | P = 0.057 | $\eta^2 = 0.02$ |
| Age × Condition | $F_{9,321} = 0.59$ | P = 0.79 | $\eta^2 = 0.02$ |
| | Strength (CFC) | | |
| Age | $F_{3,107} = 8.56$ | **P < 0.0001** | $\eta^2 = 0.19$ |
| Condition | $F_{3,321} = 53.41$ | **P < 0.0001** | $\eta^2 = 0.33$ |
| Age × Condition | $F_{9,321} = 3.17$ | **P < 0.005** | $\eta^2 = 0.08$ |

WFC, within-frequency coupling; CFC, cross-frequency coupling

**Supplementary Table 2.** Reliability of coupling strength determined within an epoch as compared to mean ICI measure averaged across the eight different epochs

| | Conditions | | | |
|---|---|---|---|---|
| Measures | REC | REO | UOT | AOT |
| | Within-frequency coupling (WFC) | | | |
| Cronbach's α | 0.986 | 0.987 | 0.984 | 0.987 |
| R | 0.973 | 0.974 | 0.968 | 0.973 |
| | Cross-frequency coupling (CFC) | | | |
| Cronbach's α | 0.942 | 0.773 | 0.901 | 0.895 |
| R | 0.891 | 0.630 | 0.821 | 0.809 |

R, correlation coefficient; WFC, within-frequency coupling; CFC, cross-frequency coupling

**Supplementary Table 3.** Reliability of network topology measures determined within two different epochs

| Measures | *Mean* | | *tSD* | | *nSD* | |
|---|---|---|---|---|---|---|
| | *Cronbach's α* | *R* | *Cronbach's α* | *R* | *Cronbach's α* | *R* |
| $S_{in}$ | 0.934 | 0.877 | 0.715 | 0.556 | 0.852 | 0.742 |
| $S_{out}$ | 0.936 | 0.879 | 0.658 | 0.490 | 0.835 | 0.716 |
| CC | 0.977 | 0.956 | 0.925 | 0.860 | 0.906 | 0.828 |
| CPL | 0.906 | 0.829 | 0.647 | 0.479 | 0.725 | 0.568 |
| $E_{local}$ | 0.901 | 0.820 | 0.772 | 0.629 | 0.789 | 0.652 |
| $E_{global}$ | 0.900 | 0.818 | 0.840 | 0.724 | 0.786 | 0.647 |

*R,* correlation coefficient; *tSD,* temporal standard deviation; *nSD,* nodal standard deviation; $S_{in}$, in-strength; $S_{out}$, out-strength; *CC*, clustering coefficient; *CPL*, characteristic path length; $E_{local}$, local efficiency; $E_{global}$, global efficiency.

**Supplementary Table 4.** ANCOVA results for the mean (M) and standard deviation (SD) across time and across nodes for the six GTA measures

| GTA measures | Factors | F-value | P-value | Partial eta squared |
|---|---|---|---|---|
| | | Mean (M) | | |
| $S_{in}$ | Age | $F_{3,105} = 20.06$ | **P < 0.0001** | $\eta^2 = 0.36$ |
| | Age × Condition | $F_{3,105} = 0.25$ | P = 0.86 | $\eta^2 = 0.01$ |
| $S_{out}$ | Age | $F_{3,105} = 19.38$ | **P < 0.0001** | $\eta^2 = 0.36$ |
| | Age × Condition | $F_{3,105} = 0.58$ | P = 0.63 | $\eta^2 = 0.02$ |
| *CC* | Age | $F_{3,105} = 4.91$ | **P < 0.005** | $\eta^2 = 0.12$ |
| | Age × Condition | $F_{3,105} = 0.96$ | P = 0.42 | $\eta^2 = 0.03$ |
| *CPL* | Age | $F_{3,105} = 15.58$ | **P < 0.0001** | $\eta^2 = 0.31$ |
| | Age × Condition | $F_{3,105} = 0.52$ | P = 0.67 | $\eta^2 = 0.02$ |
| $E_{local}$ | Age | $F_{3,105} = 8.35$ | **P < 0.0001** | $\eta^2 = 0.19$ |
| | Age × Condition | $F_{3,105} = 0.72$ | P = 0.55 | $\eta^2 = 0.02$ |
| $E_{global}$ | Age | $F_{3,105} = 15.25$ | **P < 0.0001** | $\eta^2 = 0.30$ |
| | Age × Condition | $F_{3,105} = 0.28$ | P = 0.84 | $\eta^2 = 0.01$ |
| | | Standard Deviation across time (tSD) | | |
| $S_{in}$ | Age | $F_{3,105} = 28.80$ | **P < 0.0001** | $\eta^2 = 0.45$ |
| | Age × Condition | $F_{3,105} = 1.61$ | P = 0.19 | $\eta^2 = 0.04$ |
| $S_{out}$ | Age | $F_{3,105} = 13.28$ | **P < 0.0001** | $\eta^2 = 0.28$ |
| | Age × Condition | $F_{3,105} = 2.25$ | P = 0. 087 | $\eta^2 = 0.06$ |
| *CC* | Age | $F_{3,105} = 3.85$ | **P < 0.05** | $\eta^2 = 0.10$ |
| | Age × Condition | $F_{3,105} = 1.90$ | P = 0.13 | $\eta^2 = 0.05$ |
| *CPL* | Age | $F_{3,105} = 18.18$ | **P < 0.0001** | $\eta^2 = 0.34$ |
| | Age × Condition | $F_{3,105} = 0.96$ | P = 0.42 | $\eta^2 = 0.03$ |
| $E_{local}$ | Age | $F_{3,105} = 11.85$ | **P < 0.0001** | $\eta^2 = 0.25$ |
| | Age × Condition | $F_{3,105} = 1.13$ | P < 0.34 | $\eta^2 = 0.03$ |
| $E_{global}$ | Age | $F_{3,105} = 27.89$ | **P < 0.0001** | $\eta^2 = 0.44$ |
| | Age × Condition | $F_{3,105} = 2.12$ | P = 0.10 | $\eta^2 = 0.06$ |
| | | Standard Deviation across nodes (nSD) | | |
| $S_{in}$ | Age | $F_{3,105} = 5.63$ | **P < 0.001** | $\eta^2 = 0.14$ |
| | Age × Condition | $F_{3,105} = 0.35$ | P = 0.79 | $\eta^2 = 0.01$ |
| $S_{out}$ | Age | $F_{3,105} = 4.78$ | **P < 0.005** | $\eta^2 = 0.12$ |
| | Age × Condition | $F_{3,105} = 0.14$ | P = 0.94 | $\eta^2 = 0.004$ |
| *CC* | Age | $F_{3,105} = 4.15$ | **P < 0.01** | $\eta^2 = 0.11$ |
| | Age × Condition | $F_{3,105} = 0.04$ | P = 0.99 | $\eta^2 = 0.001$ |
| *CPL* | Age | $F_{3,105} = 4.49$ | **P < 0.005** | $\eta^2 = 0.11$ |
| | Age × Condition | $F_{3,105} = 1.36$ | P = 0.26 | $\eta^2 = 0.04$ |
| $E_{local}$ | Age | $F_{3,105} = 14.12$ | **P < 0.0001** | $\eta^2 = 0.29$ |
| | Age × Condition | $F_{3,105} = 0.19$ | P = 0.90 | $\eta^2 = 0.01$ |
| $E_{global}$ | Age | $F_{3,105} = 4.64$ | **P < 0.005** | $\eta^2 = 0.12$ |
| | Age × Condition | $F_{3,105} = 0.52$ | P = 0.67 | $\eta^2 = 0.02$ |

$S_{in}$, in-strength; $S_{out}$, out-strength; *CC*, clustering coefficient; *CPL*, characteristic path length; $E_{local}$, local efficiency; $E_{global}$, global efficiency.

**Supplementary Table 5.** ANCOVA results for the temporal and nodal (positive and negative) similarity for the six GTA measures

| GTA measures | Factors | F-value | P-value | Partial eta squared |
|---|---|---|---|---|
| | | Temporal network similarity | | |
| $S_{in}$ | Age | $F_{3,105} = 5.40$ | **$P < 0.005$** | $\eta^2 = 0.13$ |
| | Age × Condition | $F_{3,105} = 2.12$ | $P = 0.10$ | $\eta^2 = 0.06$ |
| $S_{out}$ | Age | $F_{3,105} = 2.79$ | **$P < 0.05$** | $\eta^2 = 0.07$ |
| | Age × Condition | $F_{3,105} = 1.41$ | $P = 0.25$ | $\eta^2 = 0.04$ |
| *CC* | Age | $F_{3,105} = 3.42$ | **$P < 0.05$** | $\eta^2 = 0.09$ |
| | Age × Condition | $F_{3,105} = 3.11$ | **$P < 0.05$** | $\eta^2 = 0.08$ |
| *CPL* | Age | $F_{3,105} = 20.97$ | **$P < 0.0001$** | $\eta^2 = 0.38$ |
| | Age × Condition | $F_{3,105} = 2.80$ | **$P < 0.05$** | $\eta^2 = 0.07$ |
| $E_{local}$ | Age | $F_{3,105} = 1.30$ | $P = 0.28$ | $\eta^2 = 0.04$ |
| | Age × Condition | $F_{3,105} = 0.67$ | $P = 0.57$ | $\eta^2 = 0.02$ |
| $E_{global}$ | Age | $F_{3,105} = 3.95$ | **$P < 0.01$** | $\eta^2 = 0.10$ |
| | Age × Condition | $F_{3,105} = 0.87$ | $P = 0.46$ | $\eta^2 = 0.02$ |
| | | Network similarity across nodes (positive) | | |
| $S_{in}$ | Age | $F_{3,105} = 1.92$ | $P = 0.13$ | $\eta^2 = 0.05$ |
| | Age × Condition | $F_{3,105} = 0.31$ | $P = 0.82$ | $\eta^2 = 0.01$ |
| $S_{out}$ | Age | $F_{3,105} = 1.97$ | $P = 0.12$ | $\eta^2 = 0.05$ |
| | Age × Condition | $F_{3,105} = 0.01$ | $P = 0.99$ | $\eta^2 = 0.00$ |
| *CC* | Age | $F_{3,105} = 1.57$ | $P = 0.20$ | $\eta^2 = 0.04$ |
| | Age × Condition | $F_{3,105} = 0.18$ | $P = 0.91$ | $\eta^2 = 0.01$ |
| *CPL* | Age | $F_{3,105} = 0.86$ | $P = 0.47$ | $\eta^2 = 0.02$ |
| | Age × Condition | $F_{3,105} = 0.41$ | $P = 0.75$ | $\eta^2 = 0.01$ |
| $E_{local}$ | Age | $F_{3,105} = 0.91$ | $P = 0.44$ | $\eta^2 = 0.03$ |
| | Age × Condition | $F_{3,105} = 0.11$ | $P = 0.96$ | $\eta^2 = 0.003$ |
| $E_{global}$ | Age | $F_{3,105} = 1.67$ | $P = 0.18$ | $\eta^2 = 0.05$ |
| | Age × Condition | $F_{3,105} = 0.15$ | $P = 0.93$ | $\eta^2 = 0.004$ |
| | | Network similarity across nodes (negative) | | |
| $S_{in}$ | Age | $F_{3,105} = 2.09$ | $P = 0.11$ | $\eta^2 = 0.06$ |
| | Age × Condition | $F_{3,105} = 1.02$ | $P = 0.39$ | $\eta^2 = 0.03$ |
| $S_{out}$ | Age | $F_{3,105} = 1.96$ | $P = 0.13$ | $\eta^2 = 0.05$ |
| | Age × Condition | $F_{3,105} = 0.47$ | $P = 0.71$ | $\eta^2 = 0.01$ |
| *CC* | Age | $F_{3,105} = 0.13$ | $P = 0.94$ | $\eta^2 = 0.004$ |
| | Age × Condition | $F_{3,105} = 0.93$ | $P = 0.43$ | $\eta^2 = 0.03$ |
| *CPL* | Age | $F_{3,105} = 6.16$ | **$P < 0.001$** | $\eta^2 = 0.15$ |
| | Age × Condition | $F_{3,105} = 0.19$ | $P = 0.91$ | $\eta^2 = 0.01$ |
| $E_{local}$ | Age | $F_{3,105} = 3.28$ | **$P < 0.05$** | $\eta^2 = 0.09$ |
| | Age × Condition | $F_{3,105} = 1.70$ | $P = 0.17$ | $\eta^2 = 0.05$ |
| $E_{global}$ | Age | $F_{3,105} = 8.54$ | **$P < 0.0001$** | $\eta^2 = 0.20$ |
| | Age × Condition | $F_{3,105} = 0.66$ | $P = 0.58$ | $\eta^2 = 0.02$ |

$S_{in}$, in-strength; $S_{out}$, out-strength; *CC*, clustering coefficient; *CPL*, characteristic path length; $E_{local}$, local efficiency; $E_{global}$, global efficiency.

**Supplementary Table 6.** ANCOVA results for the network complexity and modular organization measures

| Measures | Factors | F-value | P-value | Partial eta squared |
|---|---|---|---|---|
| | | Complexity measures | | |
| *GE* | Age | $F_{3,105} = 11.26$ | **$P < 0.0001$** | $\eta^2 = 0.24$ |
| | Age × Condition | $F_{3,105} = 1.28$ | $P = 0.28$ | $\eta^2 = 0.04$ |
| *LE* | Age | $F_{3,105} = 27.60$ | **$P < 0.0001$** | $\eta^2 = 0.44$ |
| | Age × Condition | $F_{3,105} = 1.89$ | $P = 0.14$ | $\eta^2 = 0.05$ |
| $C_e$ | Age | $F_{3,105} = 16.60$ | **$P < 0.0001$** | $\eta^2 = 0.32$ |
| | Age × Condition | $F_{3,105} = 0.62$ | $P = 0.61$ | $\eta^2 = 0.02$ |
| $C_r$ | Age | $F_{3,105} = 11.54$ | **$P < 0.0001$** | $\eta^2 = 0.25$ |
| | Age × Condition | $F_{3,105} = 2.37$ | $P = 0.08$ | $\eta^2 = 0.06$ |
| *OdC* | Age | $F_{3,105} = 8.39$ | **$P < 0.0001$** | $\eta^2 = 0.19$ |
| | Age × Condition | $F_{3,105} = 0.42$ | $P = 0.74$ | $\eta^2 = 0.01$ |
| *PE* | Age | $F_{3,105} = 9.91$ | **$P < 0.0001$** | $\eta^2 = 0.22$ |
| | Age × Condition | $F_{3,105} = 1.93$ | $P = 0.13$ | $\eta^2 = 0.05$ |
| *CDN* | Age | $F_{3,105} = 15.99$ | **$P < 0.0001$** | $\eta^2 = 0.31$ |
| | Age × Condition | $F_{3,105} = 0.92$ | $P = 0.43$ | $\eta^2 = 0.03$ |
| *IDN* | Age | $F_{3,105} = 15.84$ | **$P < 0.0001$** | $\eta^2 = 0.31$ |
| | Age × Condition | $F_{3,105} = 1.71$ | $P = 0.17$ | $\eta^2 = 0.05$ |
| | | Modular organization measures | | |
| *Q* | Age | $F_{3,105} = 4.36$ | **$P < 0.01$** | $\eta^2 = 0.11$ |
| | Age × Condition | $F_{3,105} = 0.91$ | $P = 0.44$ | $\eta^2 = 0.03$ |
| *NofM* | Age | $F_{3,105} = 10.52$ | **$P < 0.0001$** | $\eta^2 = 0.23$ |
| | Age × Condition | $F_{3,105} = 1.72$ | $P = 0.17$ | $\eta^2 = 0.05$ |
| *nMI* | Age | $F_{3,105} = 26.25$ | **$P < 0.0001$** | $\eta^2 = 0.43$ |
| | Age × Condition | $F_{3,105} = 0.28$ | $P = 0.84$ | $\eta^2 = 0.01$ |
| *nVI* | Age | $F_{3,105} = 8.55$ | **$P < 0.0001$** | $\eta^2 = 0.20$ |
| | Age × Condition | $F_{3,105} = 0.42$ | $P = 0.74$ | $\eta^2 = 0.01$ |

*GE*, graph energy; *LE*, Laplacian energy; $C_e$, efficiency complexity; $C_r$, graph index complexity; *OdC*, offdiagonal complexity; *PE*, partition entropy; *CDN*, correlation dimension of the network; *IDN*, information dimension of the network; *Q*, modularity; *NofM*, number of modules; *nMI* = normalized mutual information; *nVI* = normalized variation of information.

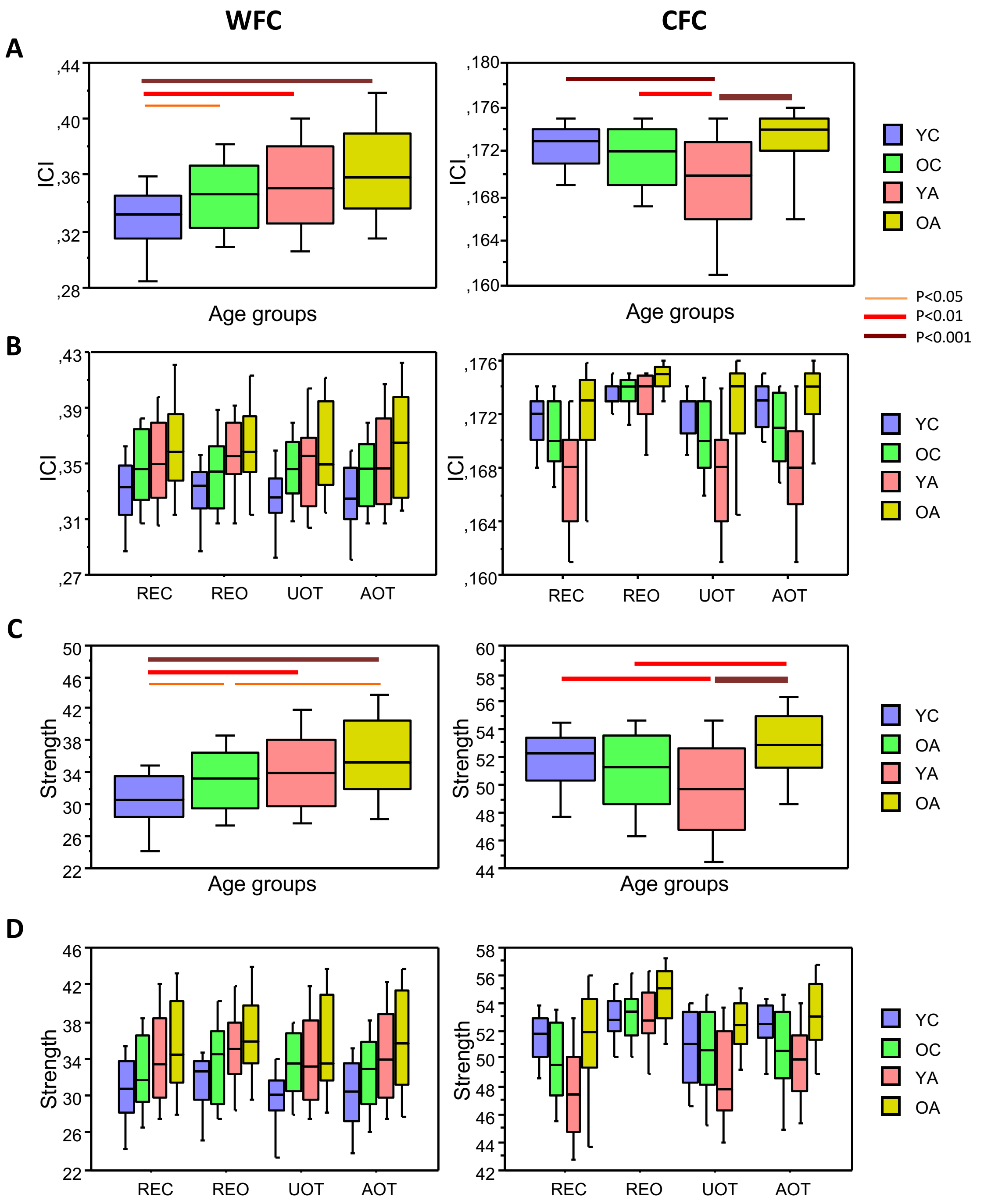


**Supplementary Figure 1. ANOVA results for WFC and CFC. (A)** Diagrams of mean *ICI* values averaged across eight 10-s segments for WFC (left) and CFC (right) across the lifespan. **(B)** Diagrams of mean *ICI* values for WFC and CFC across the lifespan under the four task conditions. **(C)** Diagrams of WFC and CFC strengths across the lifespan. **(D)** Diagrams of WFC and CFC strengths across the lifespan under the four task conditions. Age groups: YC, younger children; OC, older children; YA, younger adults; OA, older adults. Conditions: REC, rest with eyes closed; REO, rest with eyes open; UOT, unattended oddball task; AOT, attended oddball task.

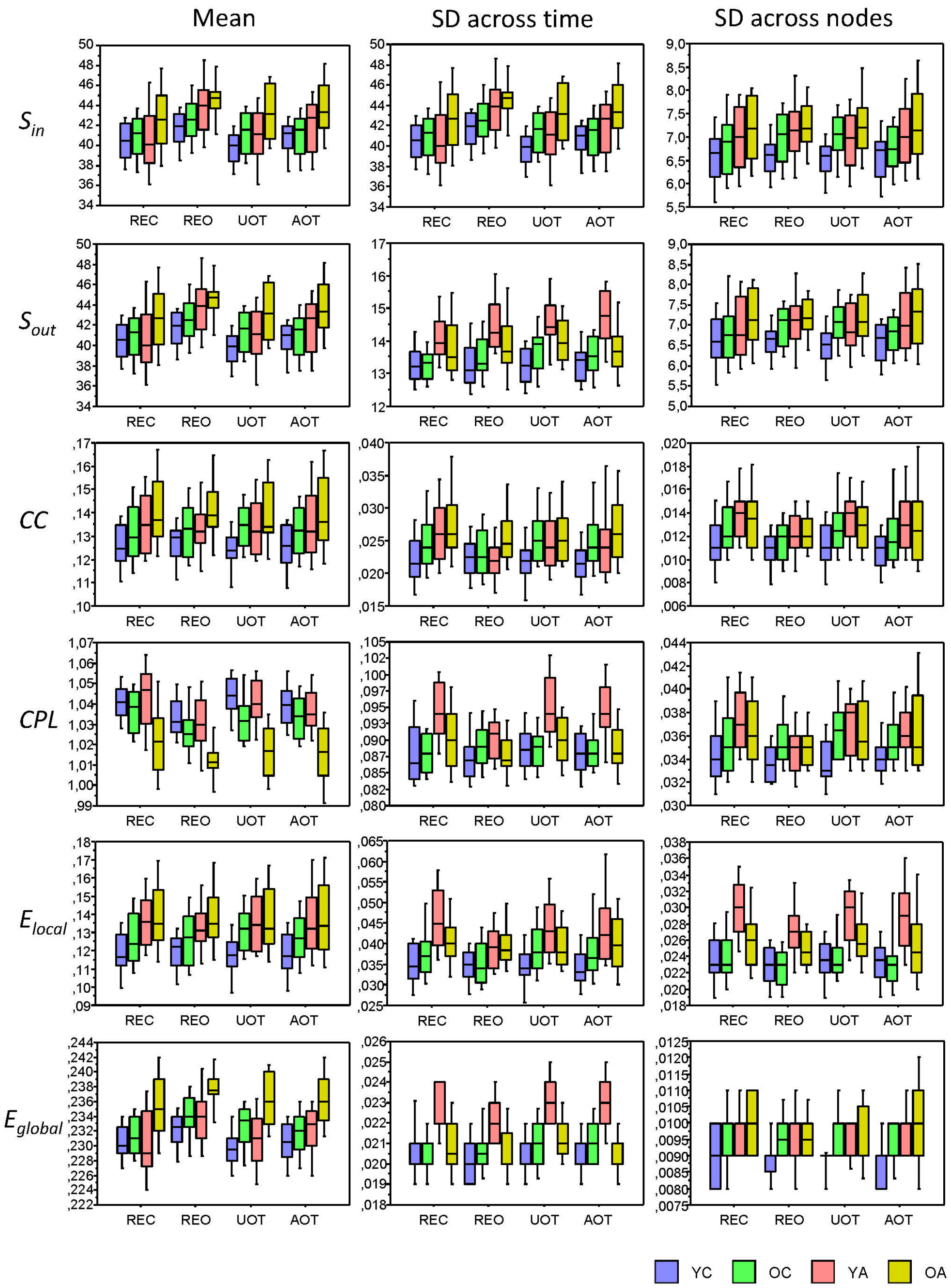


**Supplementary Figure 2. Box plots of the mean and standard deviation (SD) of the six GTA measures across the lifespan under different task conditions.** Changes of the mean and SD across time and across nodes for the six GTA measures under the different task conditions. GTA measures: In-Strength ($S_{in}$), Out-Strength ($S_{out}$), Clustering Coefficient (*CC*), Characteristic Path Length (*CPL*), Local Efficiency ($E_{local}$), and Global Efficiency ($E_{global}$). Age groups: YC, younger children; OC, older children; YA, younger adults; OA, older adults. Conditions: REC, rest with eyes closed; REO, rest with eyes open; UOT, unattended oddball task; AOT, attended oddball task.

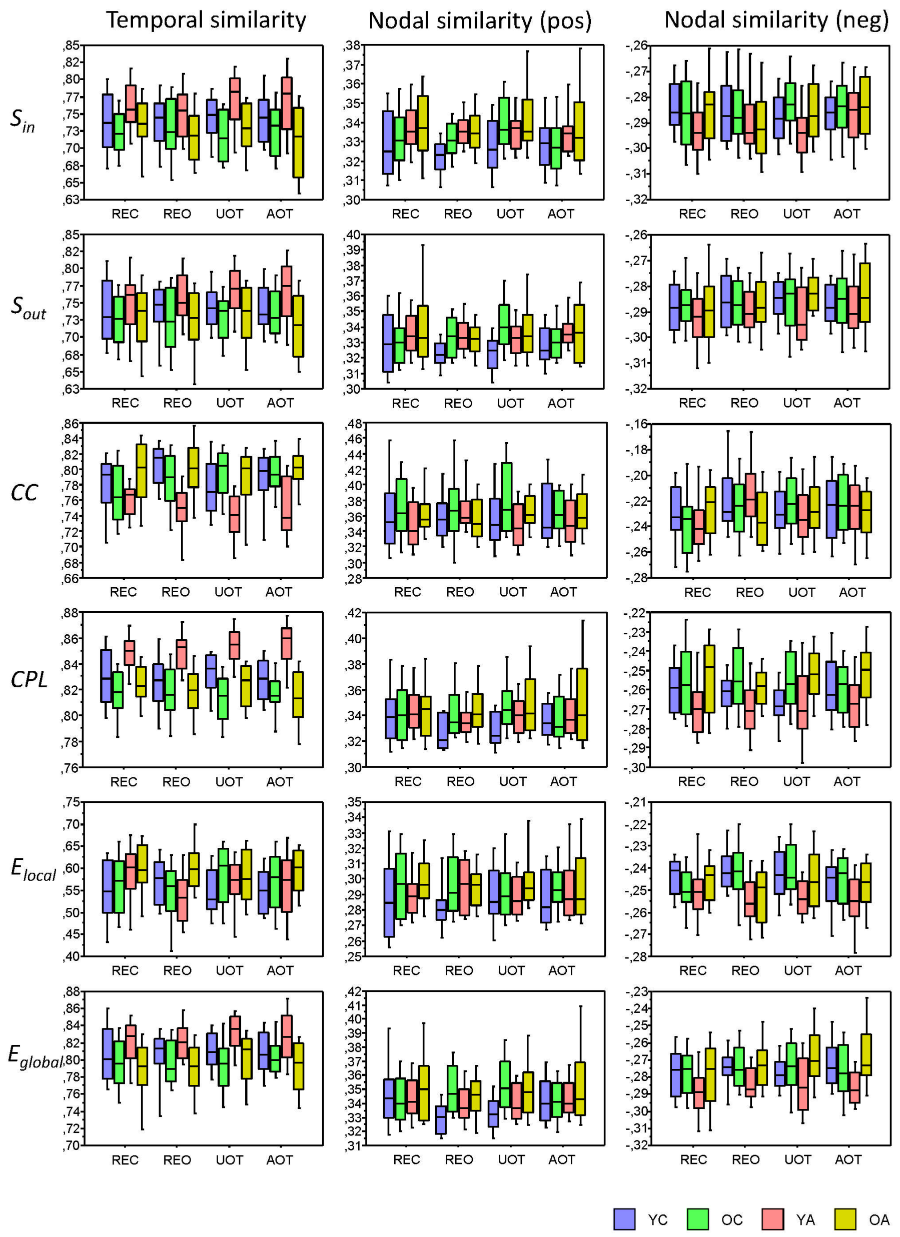


**Supplementary Figure 3. Box plots of the temporal and nodal similarity of the six GTA measures across the lifespan under different task conditions.** Temporal similarity was calculated by Pearson's product correlation between nodes among the 81 consecutive time windows, resulting in an 81 × 81 symmetric matrix. In this matrix, average strength has been determined as a *global temporal similarity index*. Nodal similarity was calculated by Pearson's product correlation between time windows among the 580 consecutive nodes, resulting in a 580 × 580 symmetric matrix. In this matrix, average strength has been determined as a *global nodal similarity index*. Since nodal network similarity contained positive as well as negative values, we calculated two means or average strengths for positive and negative correlation values, respectively. GTA measures: In-Strength ($S_{in}$), Out-Strength ($S_{out}$), Clustering Coefficient (*CC*), Characteristic Path Length (*CPL*), Local Efficiency ($E_{local}$), and Global Efficiency ($E_{global}$). Age groups: YC, younger children; OC, older children; YA, younger adults; OA, older adults. Conditions: REC, rest with eyes closed; REO, rest with eyes open; UOT, unattended oddball task; AOT, attended oddball task.

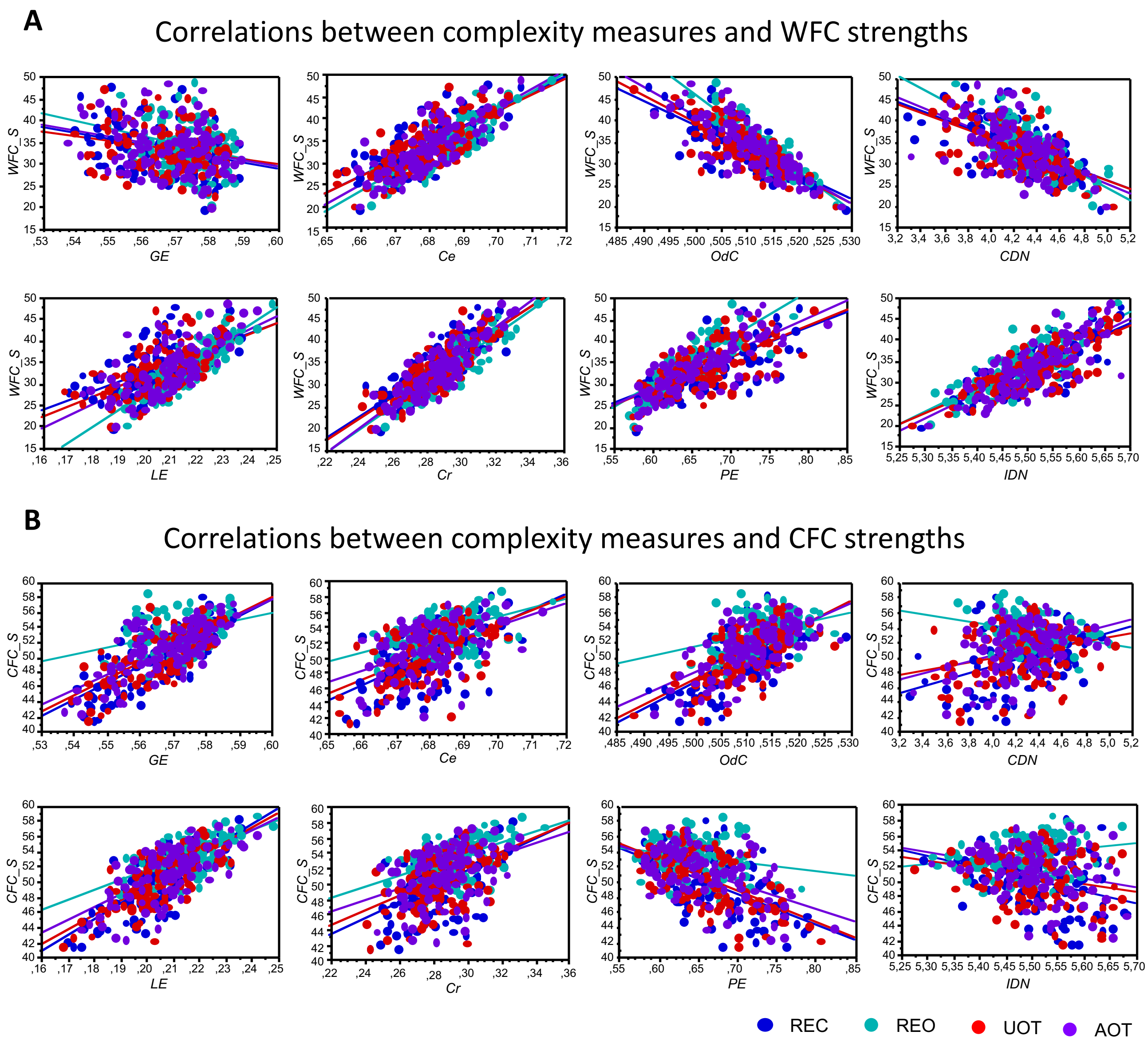


**Supplementary Figure 4. Correlation plots showing Pearson's product correlations between network complexity measures and WFC and CFC strengths. (A)** Correlations between complexity measures and WFC strengths. **(B)** Correlations between complexity measures and CFC strengths. Pearson's product correlations were calculated for each condition separately. Complexity measures: *GE*, graph energy; *LE*, Laplacian energy; $C_e$, efficiency complexity; $C_r$, graph index complexity; *OdC*, offdiagonal complexity; *PE*, partition entropy; *CDN*, correlation dimension of the network; *IDN*, information dimension of the network. Conditions: REC, rest with eyes closed; REO, rest with eyes open; UOT, unattended oddball task; AOT, attended oddball task.
